# PDL1 targeting by miR-138-5p amplifies anti-tumor immunity and Jurkat cells survival in non-small cell lung cancer

**DOI:** 10.1038/s41598-024-62064-5

**Published:** 2024-06-12

**Authors:** Fatemeh Rostami, Zahra Tavakol Hamedani, Azadeh Sadoughi, Marzieh Mehrabadi, Fatemeh kouhkan

**Affiliations:** 1 Stem Cell Technology Research Center (STRC), Iran University of Medical Science (IUMS), P.O. Box: 15856-36473, Tehran, 15856-36473 Iran; 2 Department of Biotechnology, University of Tehran, Tehran, Iran; 3 Department of Biology, Science and Research Branch, Islamic Azad University, Tehran, Iran

**Keywords:** NSCLC, miR-138-5p, PD-L1/PD-1, Jurak, A549, Calu6, Cytokine, Cancer, Immunology, Molecular biology

## Abstract

Non-small cell lung cancer (NSCLC) has constituted over 80% of the lung cancer population with a poor prognosis. Over the past decade, immunotherapy has been constructed in the enlargement of immune checkpoint inhibitors as a promising approach for NSCLC treatment. Evading the immune system using the PD-1/PD-L1 axis is an intelligent way for cancers, and T cells cannot respond fully and confront cancer. Recently, the miR-138 was reported as a PD-L1 regulator in NSCLC. However, its inhibitory impact on T-cell exhaustion has not been characterized. The present study aims to impair PD-L1 (B7-H1) expression in Adenocarcinoma cell lines using miR-138-5p and determines how it prevents Jurak cell exhaustion. To gain the purpose, first, 18 highly significant dysregulated miRNAs containing hsa-miR-138 and CD274-mRNA network were detected in NSCLC based on bioinformatics analysis. Moreover, our study revealed a high level of miR-138-5p could make significant changes like PDL1 downregulation, proliferation, and mortality rate in A549/Calu6 cells. We also simulate cancer environmental conditions by culturing Jurak cells and NSCLC cell lines under the influence of stimulator cytokines to show how miR-138-5p survives Jurak cells by targeting PD-L1/PD-1pathway.

## Introduction

Lung cancer containing Non-small cell lung carcinoma (NSCLC) and small cell lung carcinoma (SCLC) subtypes is one of the leading causes of cancer-related deaths worldwide^1^. NSCLC, which has nearly 85% of the lung cancer population, has a poor prognosis, disappointing standard therapies, and a low survival rate^2^. Thus, improving understanding of the complexity and mechanism involved in anti-tumor immunity in parallel with molecular changes could be useful in the evolution of cancer biology and tumor immunotherapy. In normal circumstances, the series of co-inhibitory and co-stimulatory receptors are involved in protecting the host against autoimmune and infectious diseases with engagement with their ligand^3,4^. Moreover, collecting proof illustrated that tumors have used these pathways as an intelligent and crucial mechanism to escape from the anti-tumor immune system and subsequently progress and metastasis^5,6^. Among those co-inhibitory pathways (receptor and ligand), programmed cell death 1 (PD-1) and programmed cell death ligand 1 (PDL1) play a vital role as an instrument in immune escaping that is used by cancer cells^6^. Interestingly, in recent decades researchers developed some drugs such as Nivolumab^7^ and pembrolizumab^8,9^ as immune checkpoint inhibitors that have blocked the PD1-PDL1 pathway. PD1(also known as CD279) is a transmembrane receptor and apoptosis-associated molecule^10^ that was known as a negative regulator of immune response by studying Nishimura and colleagues on PD1-defective mice^11^ and expressed in T cells in peripheral tissues. PD-L1 (also known as B7-H1 or CD274) is a member of the B7-H1 transmembrane receptor family encoded by the CD274 gene located on chromosome 9 in the human genome^12^, and it is generally expressed on many cell types like T cells, B cells, and epithelial cells after stimulation and activation by pro-inflammatory cytokines like IFN-γ and IL4^13^.

Furthermore, PD1 cannot be expressed in rested T cells, and it is neutralized when engaged by PDL1 in the PD1-PDL1 axis in several ways including cytokine production with substantial efficacy on IFN-γ, IL2, and TNFα production^14^. Considering the important role of the PD1-PDL1 pathway in preventing autoimmunity in normal tissues, it is not wondering that PD1 axil has operated by tumor cells to escape from immune response and finally progress and metastasize^15^. PDL1 expression has been upregulated in several tumor cells such as ovarian^16^, lung^17^, and renal cancer^18^, and it is expressed in any other cancers with an improper activation of oncogenic PI3K-AKT signaling pathway that is independent of inflammatory signals^19,20^ Conversely, PDL1 is expressed by inflammatory signals induced in other cancers, and among the cytokines, IFNγ emerges to be the most intense^21,22^. Moreover, Compelling evidence described that interaction between PD1 and PDL1 proteins in a tumor mass launched a resistance from T cell (CD8^+^)-mediated tumor cell killing by promoting T cell dysfunction, exhaustion, apoptosis, and neutralization^23^.

Over the past decades, miRNAs have been well known as small non-coding RNA regulator genes at the post-transcriptional level by targeting complementary sequences in their 3′UTR of genes, excluding mRNA translation or inducing demolition^24^.

It has been proven that miRNAs multitudinous implicate several ranges of cell functions, constantly conceived as dominant gene regulators^25^. In the present study, we investigated the association and correlations between miRNAs -mRNAs and mRNAs with related co-expression networks by the Gene Ontology term enrichment analysis as a popular method^26^ in NSCLS compared to normal samples from GEO (https://www.ncbi.nlm.nih.gov/geo). Furthermore, according to the assumed immune checkpoint inhibitory role of hsa-miR-138-5p on CD274- 3′UTR, we assessed its influential role in NSCLC, Immune escape inhibition, and Jurkat cells survival with PD1-PDL1pathway interference.

## Result

### Bioinformatics result

#### The miRNA and mRNA Samples collection

The collections of mRNA and miRNA samples related to specific subtypes of NSCLC were provided from the GEO database.

The resulting expression profile dataset by array consisted of 13,334 genes and 1556 samples, comprising 293 Normal samples, 711 adenocarcinoma (AD) samples, and 382 squamous cell carcinoma (SCC) samples as mRNA sample size.

The non-coding RNA profile dataset by array consisted of 165 miRNAs and 734 samples, including 120 Normal samples, 388 AD samples, and 141 SCC samples as miRNA sample size.

#### Dysregulated miRNAs associated with NSCLC

**Figure 1:**
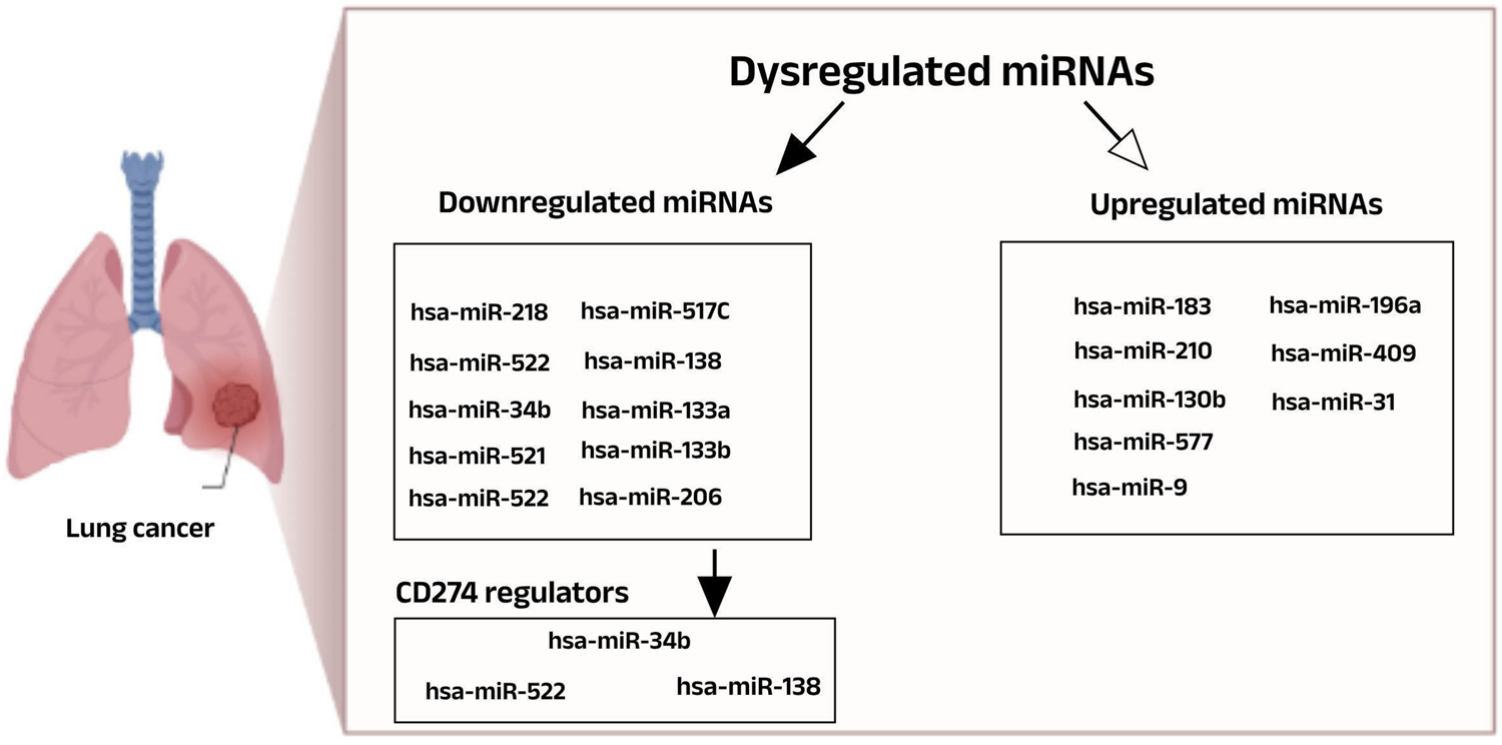
Evidence for the intersection of dysregulated miRNAs between (SCC versus normal samples) and (AD versus normal samples). The results include eight upregulated miRNAs and ten downregulated miRNAs associated with NSCLC while the hsa-miR-34b, hsa-miR-522 and hsa-miR-138 can interact with the CD274 gene.

To select the best miRNA that can interact with CD274 in NSCLC, the dysregulated miRNAs associated with NSCLC were selected using limma package in R language (version 4.1.2). The miRNAs with negative and positive Log FC are considered downregulated and upregulated miRNAs, respectively. The significance of the result was evaluated considering adjusted *P* value < 0.05, effect size > 0.2, and 95% confidence interval as the significant ranges (Table S1, S2). The result revealed downregulated and upregulated miRNAs after the intersection between the results of SCC (versus normal samples) and AD (versus normal samples). Among NSCLC-specific tumor suppressor miRNAs, hsa-miR-522, hsa-miR-34b and hsa-miR-138 can interact with the CD274 gene based on target scan database (http://www.targetscan.org/vert71) prediction (Fig. 1).

#### The mRNA samples are distributed in distinct populations separately

**Figure 2:**
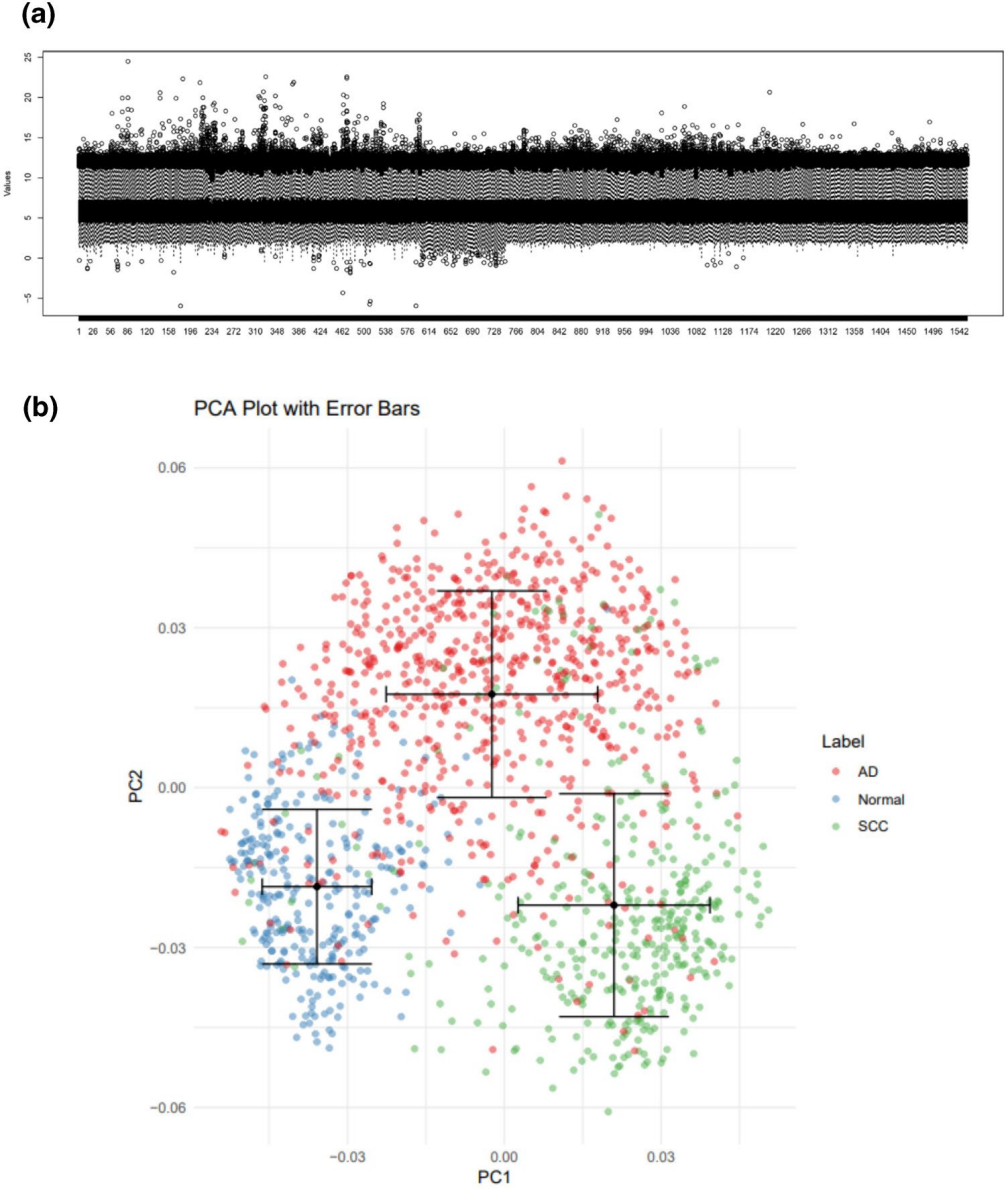
The mRNA Preprocessing and normalization. (**a**) The Normalized mRNA data are displayed on boxplot after batch-normalization. (**b**) PCA results for mRNA datasets demonstrate three populations of samples containing AD, SCC, and Normal samples that are separate from each other by labeling with red, blue, and green colors, respectively. The PCA method is utilized to capture the dataset's variability by considering these variances. The error bars displayed for each sample group indicate their respective standard deviations.

The study aims to identify three separate NSCLC sample populations containing Normal (N), adenocarcinoma (AD), and squamous cell carcinomas (SCC) samples, using an aggregated ten mRNA expression datasets. The samples show harmony after batch normalization (Fig. 2a), and the distribution of samples is shown on PC1 (first principal component) and PC2 (second principal component), which have the two most dataset variances in comparison with other principal components, Using the PCA method. These principal components capturing of the dataset's variability by considering their variances. The error bars for each sample group represent its standard deviation, as detailed in the supplementary Figure S2. Based on the PCA plot and gene's features, the samples revealed differentiation from each other in Fig. 2b (three discrete populations with blue, red, and green colors).

#### The black module revealed the most significant genes associated with NSCLC

**Figure 3:**
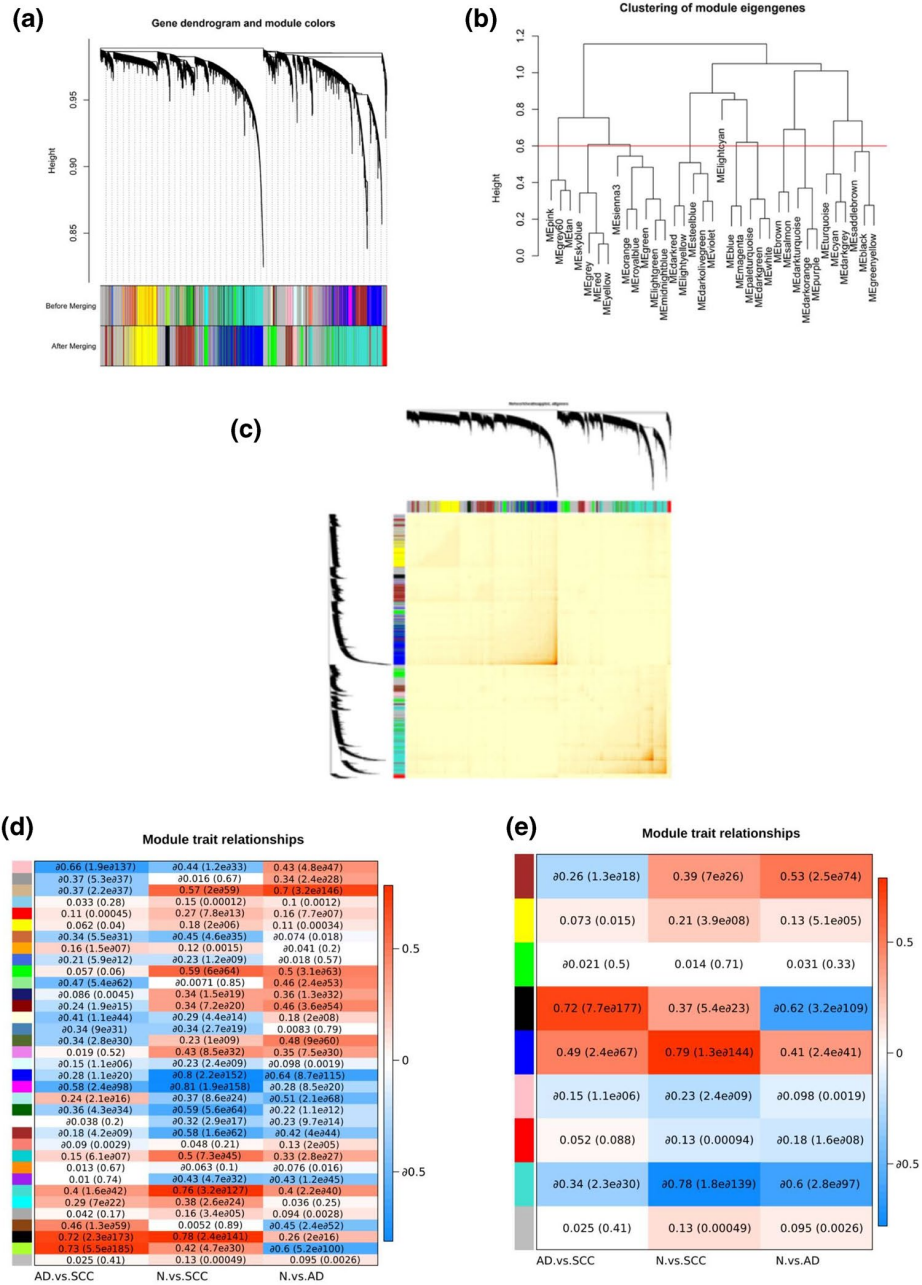
Weighted gene co-expression network (WGCNA) and module trait in NSCLC. Weighted gene co-expression network (WGCNA) identified several modules, represented by different colors. (**a**) Modules were grouped with closely linked genes and comparable expression profiles. Then the dendrogram was identified using the Dynamic Tree Cut algorithm. Module identification was accomplished with the dynamic tree-cut method, by hierarchically clustering genes with mergeCutHeight = 0.6 and minModuleSize = 30 for the resulting dendrogram. The modules (before/ after merging) were shown with specific colors at the bottom of the picture. The hierarchical clustering of the genes (**b**) and samples and a static heatmap of differentially expressed genes (DEGs) (**c**) provided valuable insight into the differences in gene expression between healthy and diseased samples. The hierarchical clustering of the genes (**b**) also revealed the similarity between modules by considering cut off that was shown with a red line. The redline showed which modules should aggregated for making larger modules. The module trait relationships were revealed before merging (**d**) and after merging (**e**). While the traits were shown on the horizontal axis, the modules were shown on the vertical axis. Trait in module-trait association determined differentiation between Normal (N) versus Adenocarcinoma (AD), Normal (N) versus Squamous Cell Carcinoma (SCC), and Adenocarcinoma (AD) versus Squamous Cell Carcinoma (SCC) samples. The positive and negative correlation revealsed upregulation and downregulation, respectively. The correlation coefficient (shown in each block) and *P* value (located in the parenthesis) were applied to show the significance of the results.

The study used the WGCNA method to analyze mRNA expression data from 10 datasets including 13,334 genes. To explore potential connections between genes, entire expression data from the datasets were imported into WGCNA without performing any preliminary screening of genes or samples allowing for a more holistic and unbiased exploration of gene expression patterns and network modules within the context of NSCLC. The soft threshold 7 was used where the following plots (scale-free topology plot and Mean connectivity plot) were bent (Figure S1). The module eigen genes and gene's Dendrogram displayed the modules before and after merging into two isolated lines based on similarity using Hierarchical clustering (Fig. 3a). Besides, the similarities between modules were revealed in a hierarchical clustering format while the red line showed where the similarity is cut off (threshold = 0.6) (Fig. 3b). The threshold = 0.6 was selected based on suitable correlation value for CD274 in the significant module. In this module, the associated genes with NSCLC and specifically CD274 were located Moreover, the correlation between modules before merging was displayed in a heat-map format (Fig. 3c).

To calculate module-trait association (MT), the comparison of different labels were considered as traits (N versus AD, N versus SCC, and AD versus SCC). The module trait association showed the correlation between a trait (located at the horizontal axis) and output modules (located at the vertical axis). The correlation coefficient and *P* value were shown in each block and the parenthesis, respectively, before merging modules (Fig. 3d) and after merging modules (Fig. 3e). Among nine modules, the black, blue, and turquoise modules containing 314, 1706, and 2441 genes showed a high correlation with significant *P* values, respectively. Moreover, the black module (selected module) showed a higher positive correlation (0.72) than other modules when comparing squamous samples versus adenoma samples. Besides, it showed downregulation in the last column (− 0.62) and low positive correlation (0.37) in the second column which showed upregulation in squamous versus normal samples. Therefore, the black module can be more confident in comparing all three types of samples (Supplementary Table S3). The CD274 was located in the black module. Thus, it was one of the most critical genes in NSCLC.

#### The mRNA-mRNA and mRNA-CD274-miRNA Networks

**Figure 4:**
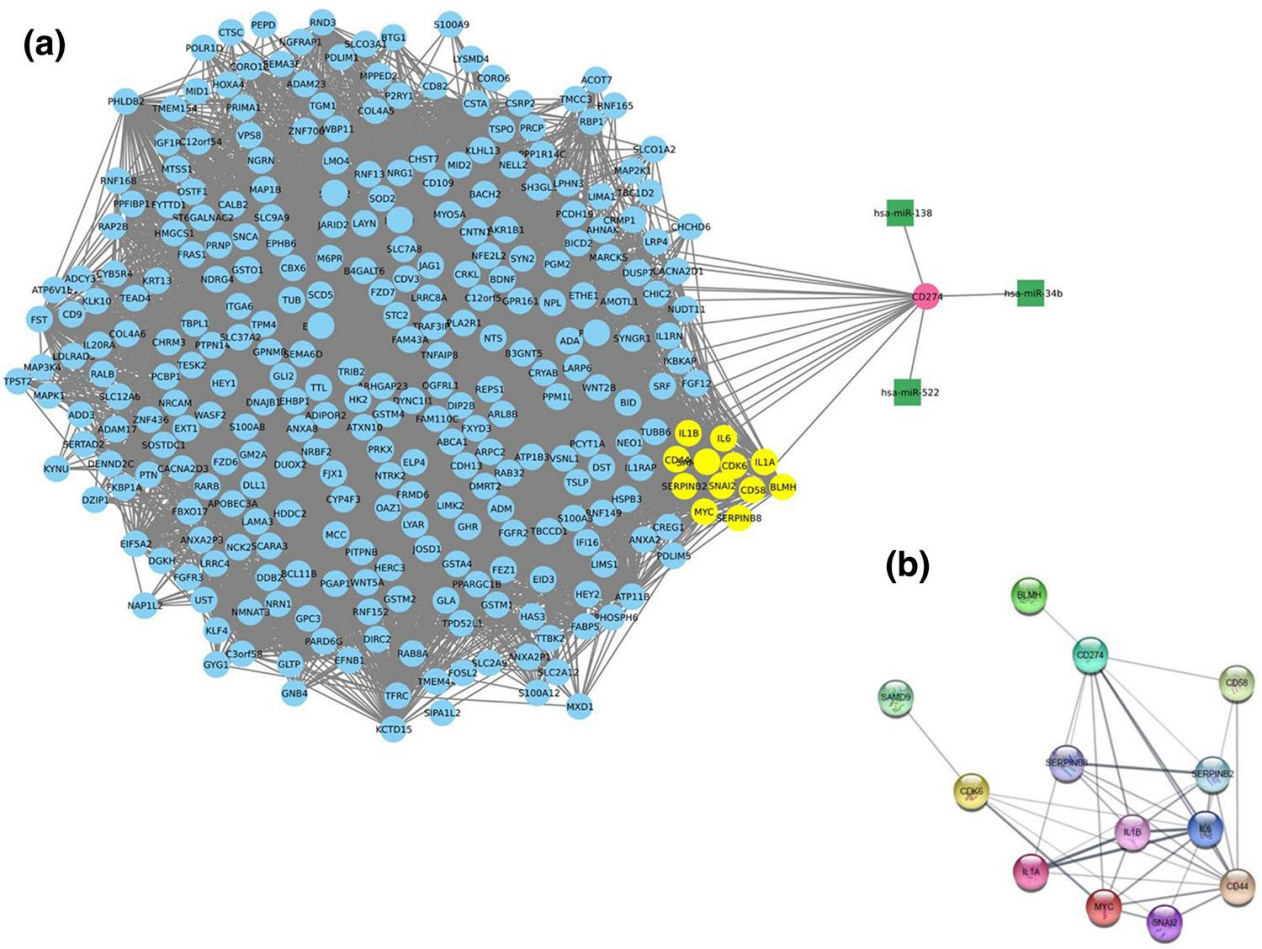
The mRNA-mRNA network constructions with CD274-associated miRNAs in the black module. (**a**) The figure reveals mRNA-mRNA interaction from the black module and miR-CD274 interaction (based on target Scan database prediction and NSCLC-associated miRNAs from the GEO dataset). The hsa-miR-138, hsa-miR-522 and hsa-miR-34b can target 3'UTR of CD274, directly. (**b**) The string output database demonstrates Genes—CD274 connection in PPI using cityscape in the black module. CD274 in the black module has close relationship with CD58, IL6, IL1B, MYC and CD44.

The significant black module containing CD274 and other genes was used for drawing the PPI network using STRING database (http://string-db.org) interactions. Among several proteins in the PPI network, the CD274 plays a key role in the PD1-PDL1. The Fig. 4b shows the close relationships between CD274 and other genes like CD58, IL6, IL1B, IL1A, MYC and CD44 and SERPINB8. The details of the statistical analysis containing exact *P* value, confidence interval and effect size for each gene based on T-test method (applied when the test statistic would follow a normal distribution) are located in Supplementary Table S4. Among NSCLC-specific tumor suppressor miRNAs, the CD274 gene and hsa-miR-522, hsa-miR-34b and hsa-miR-138 were selected to show their interaction based on bioinformatics selection (Fig. 4a). These miRNAs can downregulate CD274 by directly interaction with the 3'UTR of the CD274 gene based on the target scan database (http://www.targetscan.org/vert71). According to this prediction, while hsa-miR-138-5p belongs to miRNA families with widely conserved among vertebrates and two site seeding, hsa-miR-522 belongs to a weakly conserved family, and hsa-miR-34b has only one site seeding in CD274-3'UTR. Therefore, hsa-miR-138-5p can be a more confident candidate as a tumor suppressor miRNA for the rest of the study.

### The CD274 and hsa-miR-138 are dysregulated in NSCLC samples from GEO

**Figure 5:**
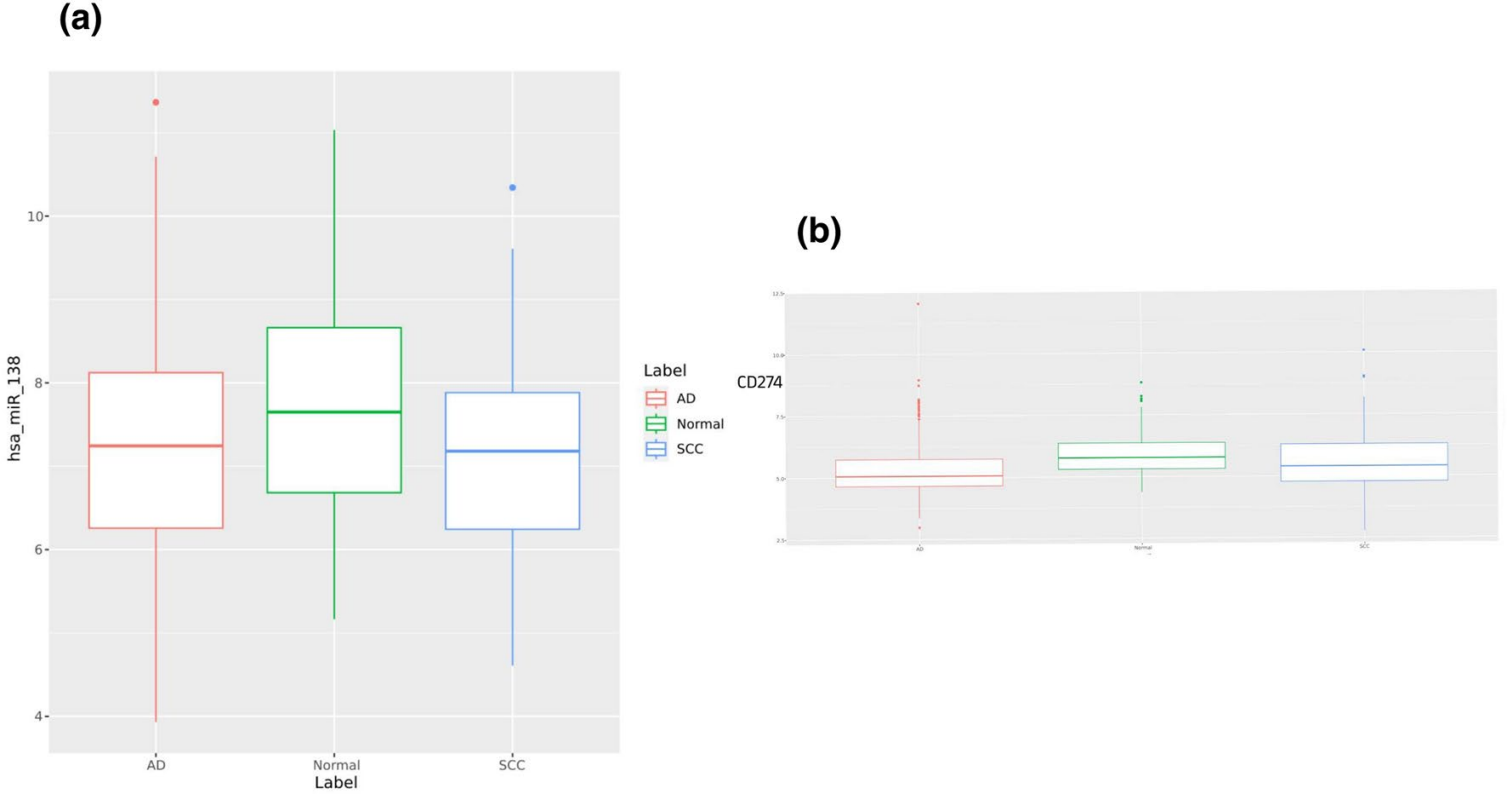
CD274 and hsa-miR-138 expression level in NSCLC subtypes. (**a**) The hsa-miR-138 expression level was calculated between three sample types (normal, adenocarcinoma, and squamous cell carcinoma). The result illustrateed that there is no significant difference between hsa-miR-138 expression level in AD and SCC subtypes (*P* value < 0.3, effect size < 0.2 and 95% confidence interval). Nevertheless, both two subtypes showed highly significant downregulation compared to normal samples (*P* value < 0.005, effect size > 0.2 and 95% confidence interval) and (*P* value < 0.0005 effect size > 0.2 and 95% confidence interval for (AD versus normal samples) and (SCC versus normal samples), respectively). (**b**) The figure demonstrated different expression levels of CD274 between two NSCLC subtypes compared to normal samples. It was expressed in the SCC subtype more than the AD subtype (*P* value < 0.05, effect size > 0.2 and 95% confidence interval).

The miR-138 expression levels on the GEO samples including NSCLC subtypes (AD and SCC) and Normal lung samples showed that hsa-miR-138 is significantly downregulated in both AD and SCC subtypes compared to normal samples (*P* value < 0.005 and *P* value < 0.0005 respectively). The effect size and confidence interval for both compared group were more than 0.2 and 95%, respectively. The expression level of hsa-miR-138 in the SCC subtype and AD subtype were not different significantly (*P* value < 0.3, effect size < 0.2 and 95% confidence interval) (Fig. 5a). The result also revealed that the CD274 expression level in the SCC subtype is more than AD subtype, significantly (*P* value < 0.05, effect size > 0.2 and 95% confidence interval) (Fig. 5b).

## Experiment result

### The miR-138-5p directly targets CD274 (PDL1)

**Figure 6:**
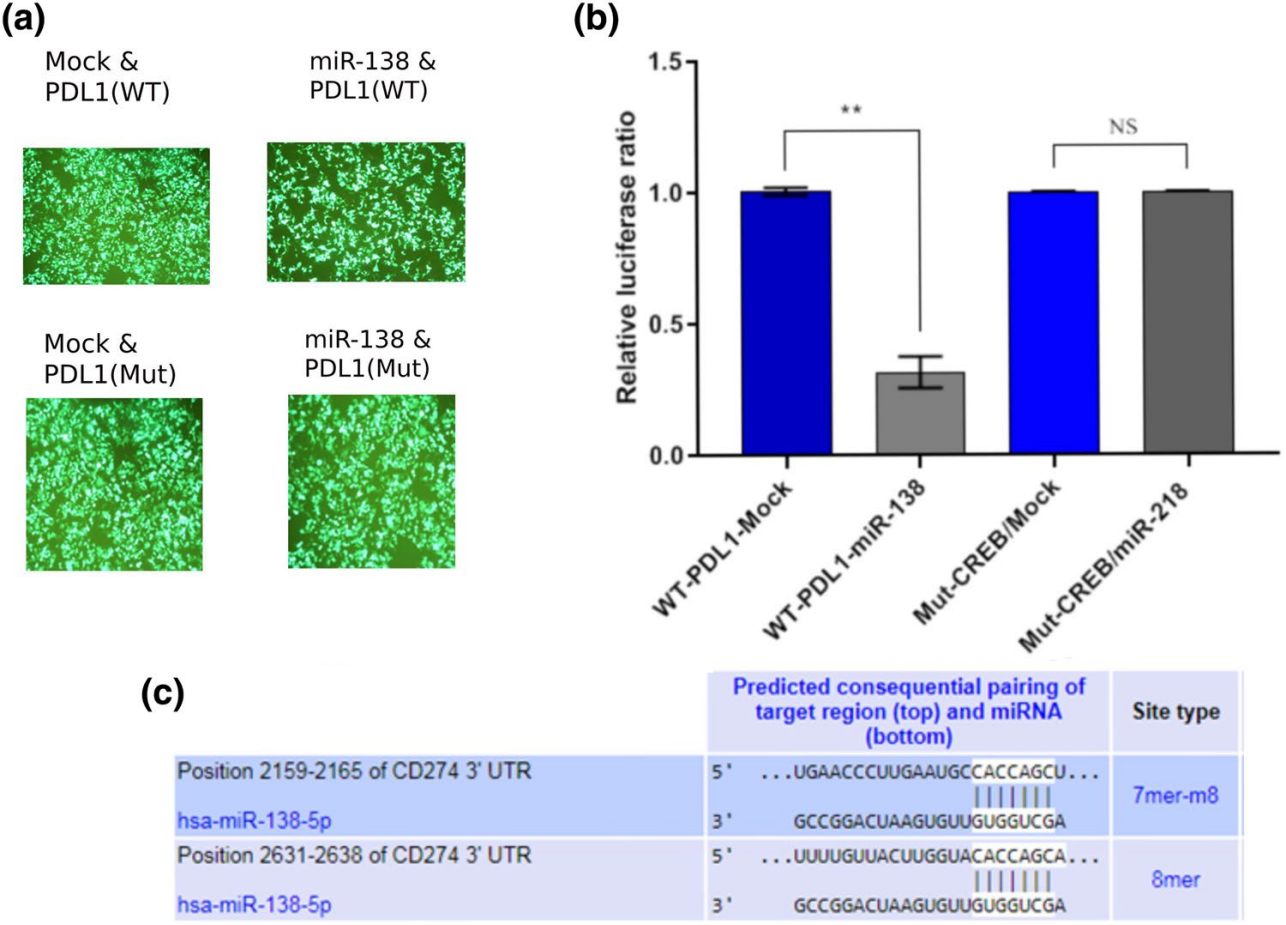
CD274 gene direct targeting with miR-138 using luciferase assay. (**a**) The efficiency of PCDH-miR-138/scramble and 3ʹUTR Wild type/Mutant co-transfection in HEK29 3T cells were visibly high in order to GFP positive cells under the fluorescent microscopy. (**b**) miR-138 directly targeted 3ʹUTR of CD274 mRNA in psiCHECK2.0 vector (**P value < 0.01) while it did not target 3′UTR mutant type, significantly. (**c**) Predicted 7mer and 8mer conserved binding sites of miR-138-5p on CD274-3ʹUTR mRNA from the Target Scan database showed a strong interaction between miR-138 and CD274.

To confirm whether miR-138 directly targets 3ʹUTR of CD274, the relative luciferase assay was performed. The luciferase activity was significantly decreased by 67% in miR138-3′UTR co-transfected cells in comparison with scramble-3ʹUTR transfected cells (***P value < 0.001) (Fig. 6). This result approved direct targeting of the CD274 (B7H1) by miR-138-5p. Therefore, exclusive interaction between miR-138-5p and the distal region of CD274-3′UTR coding mRNA would ensure suppression of NSCLC progression, and it would be effective for interrupting the PD1-PDL1 interaction.

### Transfection and transduction

**Figure 7:**
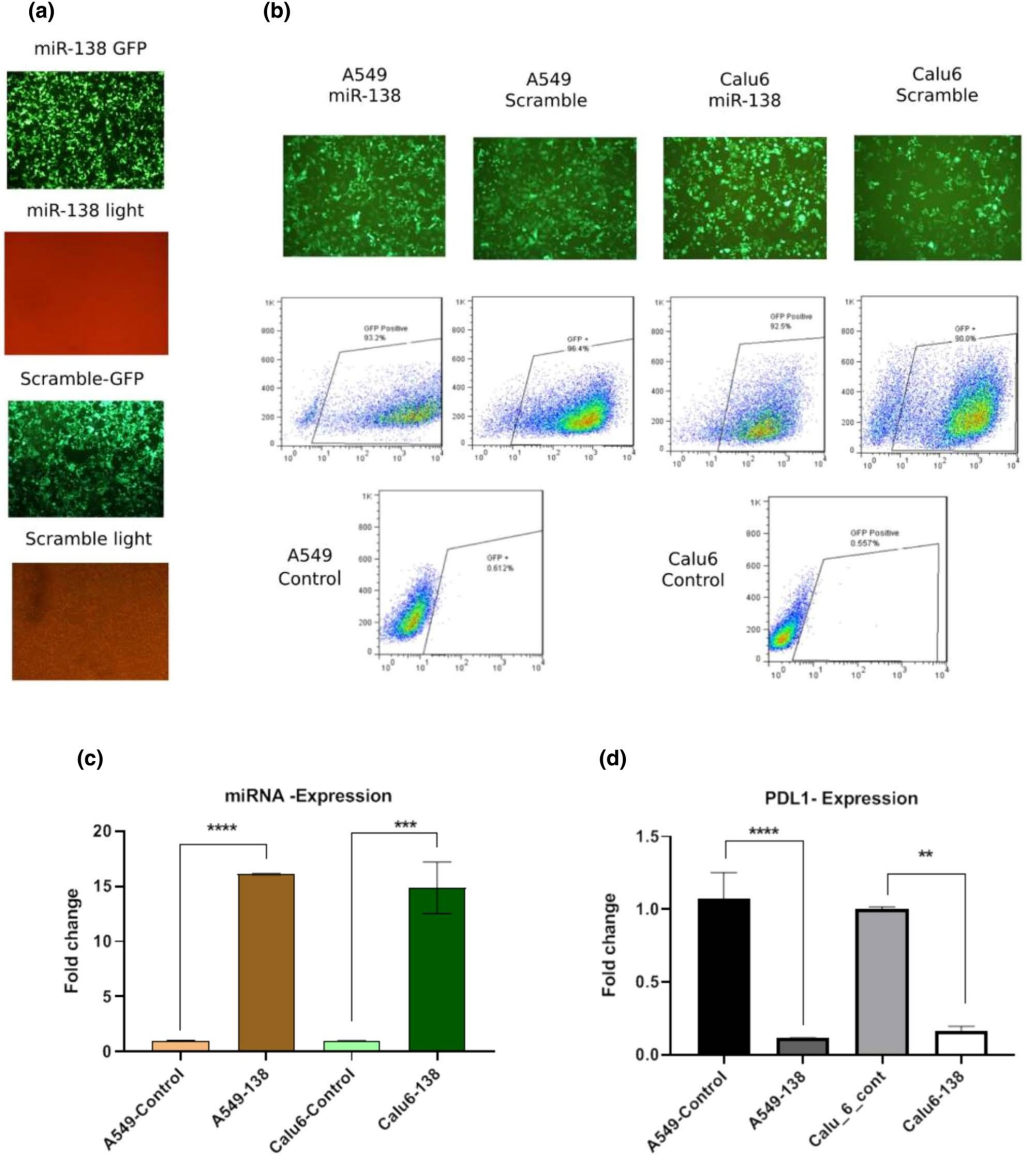
Results of HEK transfection, lentivirus transduction and relative PDL1 and miR-138-5p mRNA expression level in different groups using qRT-PCR. (**a**) The result of Co-transfection of miR-138-5p /scramble with lentiviral helper vectors revealed over 90% GFP + in HEK293T cells using fluorescent microscopy. (**b**) The efficiency of transduction was confirmed in both A549 and Calu6 cells using fluorescent microscopy and flow cytometry. The flow cytometry results proved over 90% GFP + transduced cells population compared with control cells. (**c**) The miR-138-5p expression was considerably increased by 16- (****P value < 0.0001) and 14-fold (***P value < 0.001) when A549 and Calu6 cells were transduced by recombinant miR-138-5p lentivirus, respectively. (**d**) The overexpression of miR-138-5p induced a decline in the CD274 mRNA level in transduced A549 (****P value < 0.0001) and Calu6 (**P value < 0.01) in comparison with scramble.

To produce the NSCLC cell lines that expressed miR-138, the lentiviruses carried miR-138 were constructed. To construct lentiviruses, HEK cells were co-transfected with PCDH-miR-138 and helper vectors (Fig. 7a). The treatment group and control group were provided by transducing A549 and Calu6 cells with the PCDH-miR-138 vector and the PCDH-scramble vector, respectively. The PCDH vector contained the GFP (Green fluorescent protein) coding gene, which allowed for observation of the transduced cells through fluorescent microscopy if the transduction procedure was successful and the expression genes were functioning properly. The Fig. 7b shows the appearance of the transduced NSCLC cell lines under fluorescent microscopy. Moreover, the percentage of GFP + cells was determined using flow cytometry (over 90% population). This purity covered the more significant results for the rest of the study.

### miR 138-5p overexpression modulates CD274 expression level in NSCLC cell lines

To confidence that the transduced NSCLC cell lines overexpressed miR-138 and downregulated CD274 (PDL1), the Real-Time PCR was performed. The overexpression of miR-138-5p was demonstrated by 16- and 14-fold in A549/Calu6- miR-138-5p transduced cell lines (**P value < 0.01) in comparison with scramble as a control group, respectively (Fig. 7c). The results showed that PDL1 mRNA expression levels significantly decreased in A549 (89% downregulation, *P* value < 0.0001) and Calu6 (80% downregulation, *P* value < 0.01) following miR-138-5p overexpression (Fig. 7d).

### The miR-138-5p induced apoptosis and declined proliferation in A549 and Calu6

**Figure 8:**
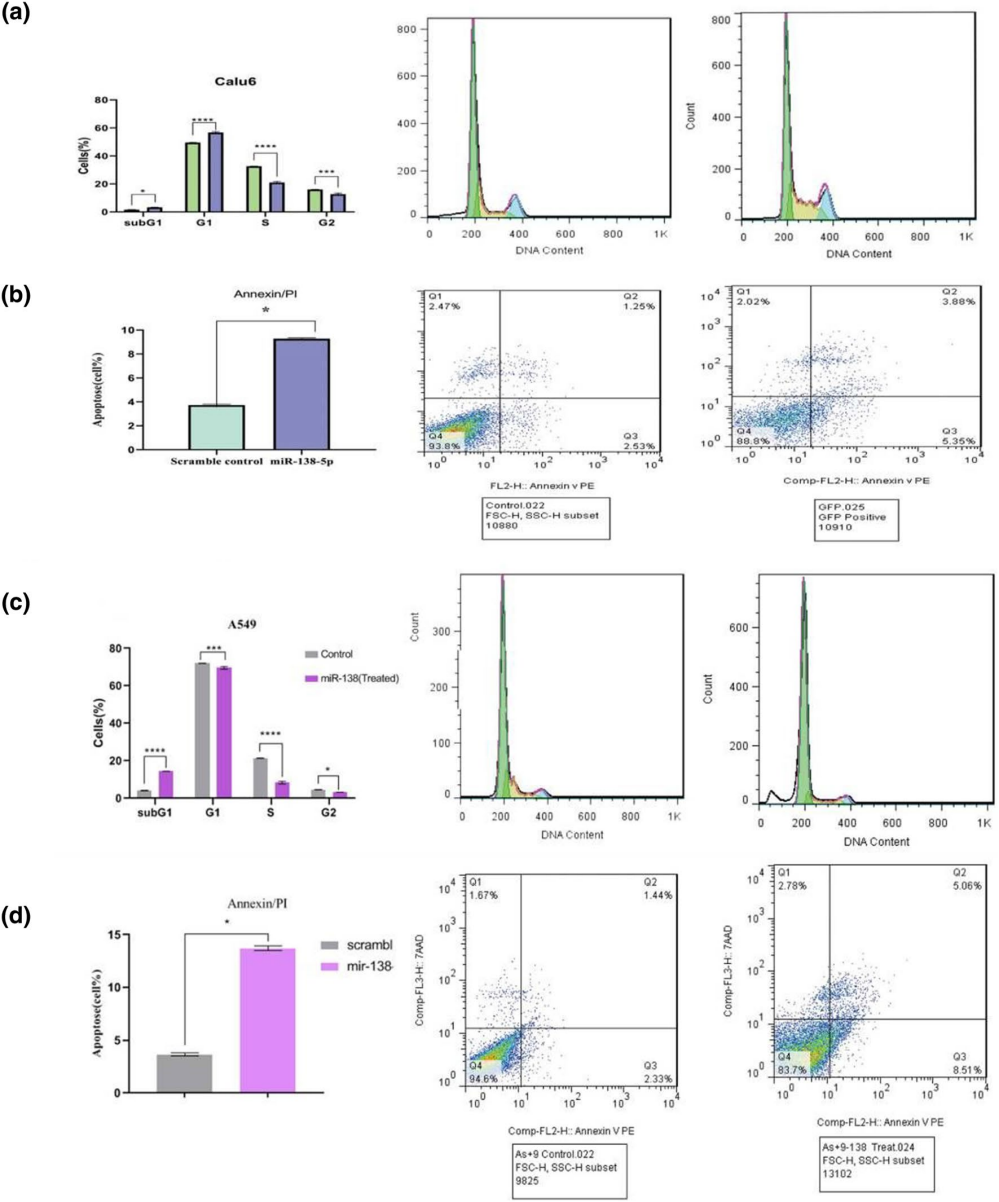
Impact of miR-138 overexpression on cell cycle and apoptosis in NSCLC cell lines**.** Cell cycle analysis by PE staining and flow cytometry in (**a**) Calu6 cells revealed a significant increase in sub-G1and G1 phases and a decrease in S and G2 phases (**c**) A549 cells demonstrated an increase in subG1phase and a decrease in G1, S, and G2 phases after transducing with miR-138-5p compared to scramble group. The histograms of annexin/PE analysis showed an increase in the percentage of apoptotic cells in (**b**) Calu6 and (**d**) A549 cell lines after transduction with miR-138-5p and scramble group. The star above the bars (*P value < 0.05), (*P value < 0.05), (***P value < 0.001), and (****P value < 0.0001) represents a significant difference concerning the related control.

To determine the effect of miR-138-5p on apoptotic and proliferation of the NSCLC cell lines, the A549/Calu6 cells (control) and transduced miR-138-5p A549/Calu6 cells were analyzed by comparing cell cycle phases and apoptotic assay using flow cytometry. The result of Fig. 8 illustrated that the miR-138-5p overexpression led to arrest in S and G2M phases (****P value < 0.0001 and ***P value < 0.001, respectively), significantly. Meanwhile, the sub-G1 and G0-G1 parts sharply increased in the transduced miR-138-5p group compared to the scramble group (control) (*P value < 0.05, ****P value < 0.0001, respectively). The relative flow cytometry histograms displayed the population of early and late apoptotic cells in the Q3 and Q2 quadrants, respectively. This data illustrated that the miR-138-5p overexpression led to a slight increase in the percentage of both early and late apoptotic cells in 72h after transduction (around 7%), and this time could be confident for co-culture assay.

### The expression level of PDL1 and PD1 overexpressed due to cytokine stimulation

**Figure 9:**
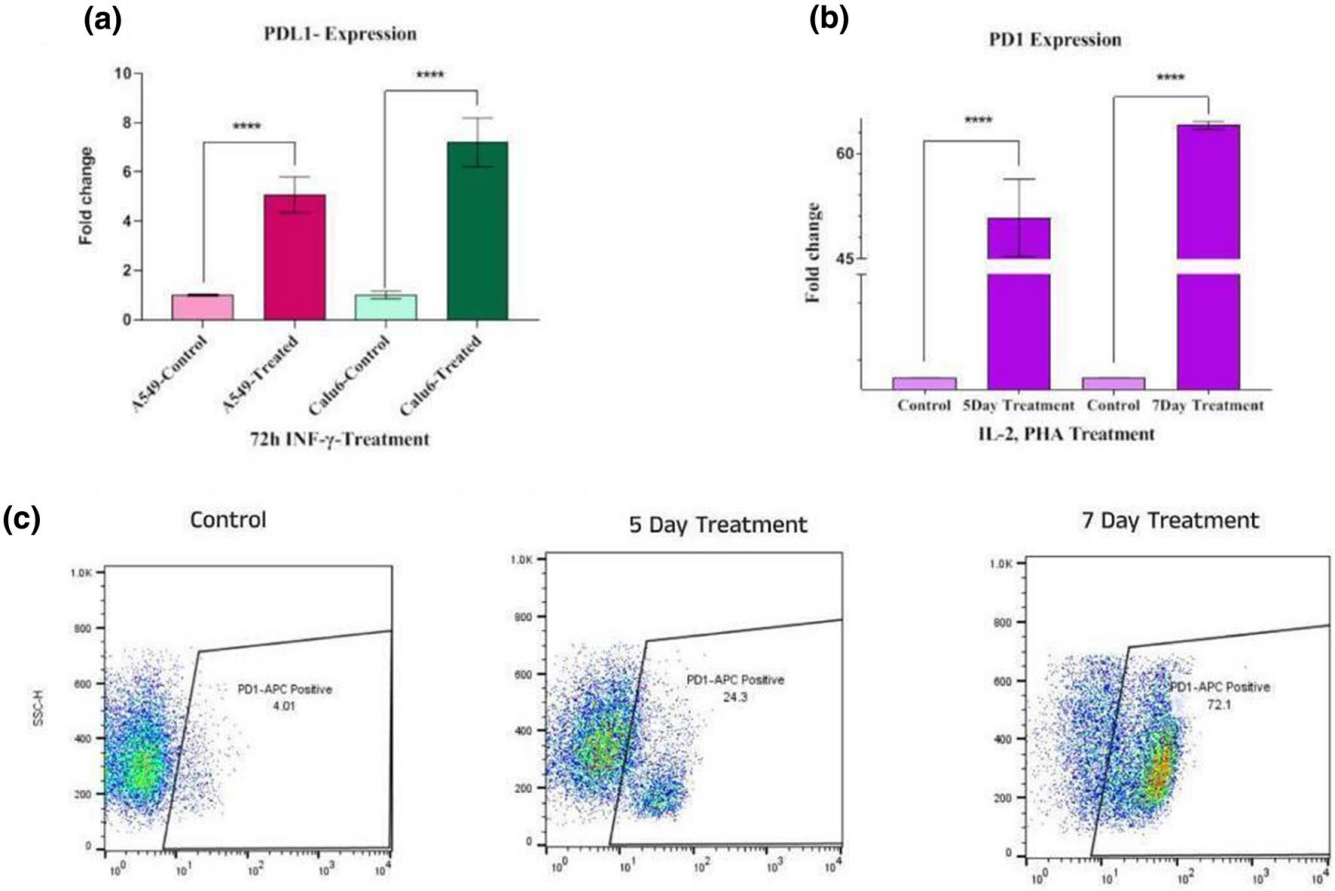
The cytokines act as stimuli to induce PD1 and PDL1 expression. (**a**) IFN-γ treatment for 3 days stimulated A549/Calu6 cells by increasing PDL1 expression (fivefold and sevenfold) significantly (****P value < 0.0001). (**b**) The relative expression level of the CD279 gene (PD1) increased significantly when Jurak cells were stimulated with IL-2 and PHA in 5 and 7 days (****P value < 0.0001). The star above the bars represents a significant difference according to the relevant control authority. (**c**) The diagrams show the percentage of PD1 protein expression on the surface of the stimulated Jurak cells (treated cells with PHA and IL2 after 5 and 7 days) that were stained with APC compared to the control group). The diagram illustrates that stimulation of the Jurkat cell line with stimulator cytokines (PHA and IL2) for 7 days by 72.1% PD1posotive population was far more beneficial than 5-day stimulation.

To obtain the high-efficiency protein expression, the A549/Calu6 and Jurkat cells were stimulated with IFN-γ and (PHA and IL-2), respectively. The PDL1 expression was meaningfully over-expressed in mRNA levels in A549 and Calu6 cell lines (fivefold and sevenfold, respectively) after culturing with IFN-γ in 3 days (Fig. 9a). Besides, to attain a high expression level of PD1 on Jurkat cells, first, the same treatment was considered for different times (5 days and 7 days) in separate wells, and both samples were assayed with Real-Time PCR and flow cytometry. According to our Real-Time PCR result, the mRNA expression level of PD1 increased remarkably (64-fold and 50-fold) due to treatment for 5 and 7 days, respectively (Fig. 9b). The results of gene expression were calculated after normalization with HPRT1 mRNA normalizer (for CD274 and CD279), and SNORD47 as miRNA genes normalizer using the 2^−ΔΔCt^ method. According to flow cytometry results, the percentage of PD1-positive cells was 24% for 5 days and 72.1% for 7 days (Fig. 9c). As a result, the optimum expression of PD1 at mRNA and protein levels was obtained within 7 days of treatment. Therefore, the miR-138-5p played a critical role in reinforcing the immune system and controlling cancers.

### The miR-138-5p inhibits immune escape by targeting PDL1 in A549 and Calu6 cell lines and surviving Jurkat cells

**Figure 10:**
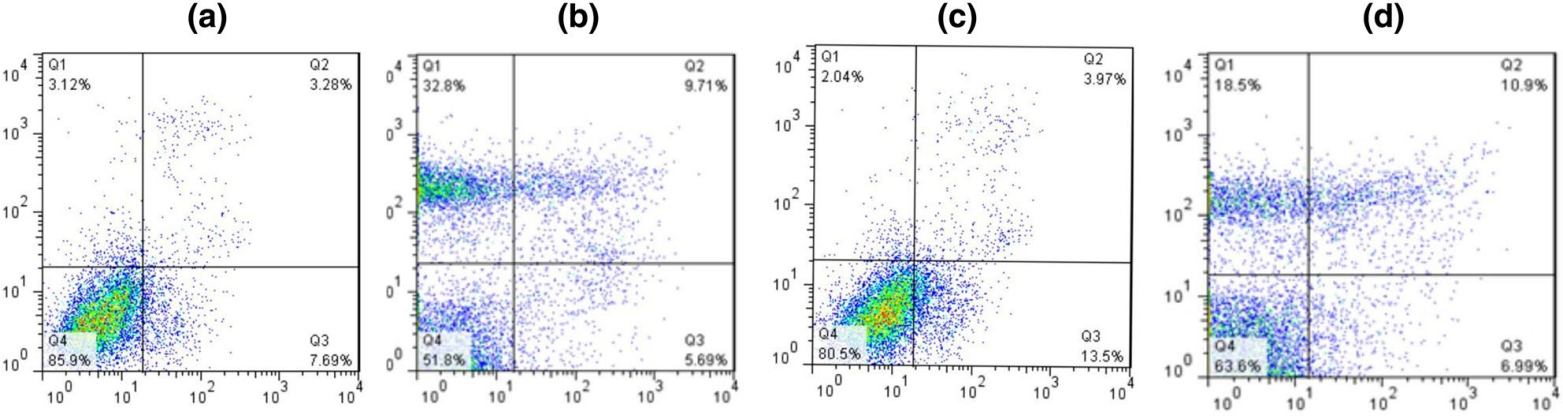
Cell apoptotic population of Jurkat cells after co-culturing. The diagrams indicate analysis apoptosis of Jurak cells using Annexin V/7AAD double staining approach after co-culturing with (**a**) Calu6-miR-138-5p (**b**) calu6-scramble (**c**) A549-miR-138-5p (**d**) A549-scramble. The apoptotic cells include four distinct populations, which can be considered Q1 (Necrosis), Q2 (Late Apoptosis), Q3 (Early Apoptosis), and Q4 (Live Cells) based on the gradual loss of red staining. The percentage of alive jurkat cells was 51.8% when they co-cultured with Calu6 (control group) (**a**) while their alive population was 85.9% when they co-cultured with miR-138-5p-transduced Calu6 cells (**b**). Meanwhile, the percentage of alive jurkat cells after co-culturing with A549 (control group) and miR-138-5p-transduced A549 cells were 63.6% (**c**) and 80.5% (**d**), respectively.

To investigate how miR-138-5p has a positive effect on the Jurkat cell's survival, the transduced/control Calu6/A549 cells were co-cultured with the Jurkat cell line in a separate flask. All adherent and suspended cells were stimulated with specific cytokines before the co-culture condition. The result revealed a remarkable decline in mortality among the Jurkat cell population when they co-cultured with miR-138-5p-transduced A549/Calu6 cells in comparison with the control groups (Fig. 10). To gain an accurate result, the percentage of alive Jurkat cells that co-cultured with miR-138-5p-transduced cells (Fig. 10a/c) was subtracted from the percentage of lived Jurkat cells in relative control groups (Fig. 10b/d). Among the cell lines, the miR-138-5p have more effect on the function of Calu6 cells than A549 cells as it inhibits around 34.1% and 16.9% Jurkat cells mortality, respectively, when the Jurkat cells co-cultured with miR-138-5p- transduced calu6/ A549 cells compared to relative control groups.

## Discussion

The PD1-PDL1 pathway is a clever mechanism used by tumor cells to evade the immune system and exhaust T cells in environmental tumors. To counter this, we aimed to interrupt this axis by targeting PDL1 in NSCLC cell lines and prevent T cell apoptosis and exhaustion in the tumor environment. To prove our hypothesis, we began by identifying miRNAs and mRNAs that are significantly dysregulated in NSCLC, using over 1300 samples from GEO and various bioinformatics methods. As a result, we discovered 18 miRNAs that are significantly dysregulated in NSCLC, with ten of them significantly downregulated and eight of them significantly upregulated. We also found the significant module, which detected the genes that are dysregulated in NSCLC samples compared to Normal samples from GEO, using the WGCNA method. Besides, CD274 was significantly upregulated in NSCLC samples. It proves that CD274 plays a key role in NSCLC. Among dysregulated miRNAs, we determined which miRNAs as suppressor-mRNA directly targeted CD274 and prevented PDL1 expression. Finally, we identified miR-138 as an effective tumor suppressor miRNA in NSCLC that can interrupt the PDL1-PD1 axis and prevent Jurak cell (T cell) apoptosis. To test our hypothesis, we simulated the tumor environment by co-culturing stimulated control and miR-138-treated NSCLC cell lines with a stimulated Jurket cell line. The results showed that miR-138 plays an essential role as a PD1-PDL1 regulator and Jurak cell survivor.

In the present study, we identified 18 dysregulated miRNAs including 10 downregulated and 8 upregulated miRNAs (Fig. 1) associated with NSCLC. Among upregulated miRNAs, miR-19a, miR-196a and miR182 have been known as prognosis markers because of their expression in early-stage cancers^27^. Specifically, in other studies, miR-182 and miR-19a were found in 3 subtypes of NSCLC, but not in the SCC classification^28^. In parallel with our result that dedicated miR-577 to the upregulated group, recently, Zhang and colleagues reported the introduced a potential interactor element for downregulating miR-577 in A549 and DDP cell lines of NSCLC^29^. Moreover, current study, in 2023, suggested miR-210 could serve as a potential therapeutic target for the treatment of lung adeno carcinoma^30^. The current studies have reported the tumor suppressor role of miR-130b and miR-409-3p in NSCLC while they have oncogenic role in other cancers. For instant, miR-130b has oncogenic role in leiomyosarcoma while it has tumor suppressor role in lung cancer^31,32^. Furthermore, although the miR-409-3p revealed both low expression and high expression level in NSCLC patients, it had affected on their overall survival by inhibiting cancer progress, metastasis and invasion^33–35^.

Among the downregulated group, except miR-206/133b cluster that has been reported both tumor suppressor and oncogenic roles in NSCLC^36,37^, other downregulated miRNAs have had tumor suppressor roles in several cancers including NSCLC^38–46^. However, the role of miR-517c in lung cancer has been blurred. Therefore, future studies can clarify new aspects of the relationship between this miRNA with NSCLC.

To investigate which genes are associated with NSCLC, the WGCNA approach to construct the CD274-mRNA co-expression networks after significant module detection was used. According to our lateral Bioinformatics results, CD274 has a close relationship with some genes like CD44, CD58, IL6, IL1A, IL1B, and MYC in NSCLC (Fig. 4) samples that have been confirmed in current investigations. For instance, in 2019, Kong and colleagues demonstrated that CD44 promotes the expression of PDL1 in lung and breast cancers^47^. Additionally, a separate study identified CD58 as an inducer of PDL1 expression at the protein level in melanoma tissue samples^48^. In NSCLC, MYC was found to regulate the expression of CD274 (PD-L1) independently of HIF, leading to immunosuppressive effects and promoting tumor growth^49^. IL6 has also been shown to promote PD-L1 expression and immune escape in pancreatic and renal cells^50^, and IL6 signaling can lead to resistance to anti-PDL1 cancer immunotherapy^51^. Recent studies suggest that IL-1A may play a significant role in the induction of an immunosuppressive tumor microenvironment, and increase PDL1expression that was in line with our bioinformatic result^52^. A study investigating melanoma found a positive correlation between tumor PD-L1 expression and serum IL-1B^53^. In future studies, researchers can use our bioinformatics results to explore the regulation of CD274 through mRNA-mRNA interactions with IL-1A, IL1-B, IL-6, and CD58 in NSCLC, providing a novel standpoint for further research.

Several studies demonstrate that the PD-1/PD-L1 axis plays a critical role in the regulation of T cell exhaustion Because of chronic exposure to high levels of PD-L1. For example, a study by Zou et al.^54^ showed that chronic exposure to PD-L1 leads to the upregulation of PD-1 and other inhibitory receptors on CD8^+^ T cells, which impairs their ability to control tumor growth. Therefore, using some inhibitors like miRNAs for interrupting PD-1/PD-L1 would be imperative. Among 10 NSCLC-specific tumor suppressor miRNAs based on the target scan dataset, miR-138-5p would be the best candidate for suppressing PD-1/PD-L1 and consequently T cell exhaustion and apoptosis (Fig. 6c). For this reason we were curious what is the expression level of mir-138 in the NSCLC samples. In this section of the bioinformatics methods, we investigated the expression levels of CD274 and miR-138-5p in normal and NSCLC samples from the GEO database, including AD and SCC subtypes. As shown in Fig. 5, miR-138-5p was found to be downregulated in both subtypes, while the expression level of CD274 mRNA was higher in the SSC subtype than in the AD subtype. Current study has demonstrated that miR-138-5p can downregulate PD-L1 expression through 3'UTR targeting in human colorectal cancer^55^. Our luciferase assay confirmed that miR-138-5p could inhibit PDL-1 by 67% and significantly reduce its level in miR-138-transduced cells (Fig. 6).

Given our findings from the GEO database, which showed a low expression level of miR-138-5p in both SCC and AC samples compared to normal samples (Fig. 5), we overexpressed miR-138-5p by transduced and selected NSCLC cell lines with viruses carrying miR-138-5p (treated) and scramble (control) (Fig. 7). Our results showed that miR-138-5p overexpression (16-fold for A549 and 14-fold for Calu6) contributed to PDL1 downregulation in A549 and Calu6 cell lines by 89% and 80%, respectively (Fig. 7).

Some previous studies have shown that miR-138-5p plays a role in inhibiting proliferation, migration, and metastasis and inducing apoptosis in various cancers, including gastric, glioma, colorectal, and lung cancer^56–59^. However, most studies on NSCLC have focused on the A549 cell line^60,61^, so we designed an experiment to compare the behavior of A549 and Calu6 cell lines under the effect of miR-138-5p. Our study involved experiments to validate the impact of miR-138-5p on PDL1 expression, cell cycle, and apoptosis in adenocarcinoma cell lines, specifically A549 and Calu6. It also induced apoptosis by increasing the percentage of dead cells and decreasing proliferation in both NSCLC cell lines after 48 h of transduction, but it was more effective in A549 cells than Calu6 (Fig. 8).

The miR-138, which functions as a tumor suppressor miRNA, not only regulates proliferation but also has a close relationship with immune response. It directly targets PD1 and PDL1 in DC and T cells in NSCLC^62–64^. Although the efficacy of working on PD-L1 has been more efficient to date^61^, the PD-1/PD-L1 axis is mysteriously heterogeneous, with the effective rate of PD-L1 expression being approximately 40% in melanoma and lung cancer samples, and tumor samples being divided into PDL1 positive and PDL1 negative^61,63^. Additionally, a study has noted that the PD-L1 expression in SCC was significantly higher than non-squamous NSCLC (AD and other histological NSCLCs)^65^. Therefore, our significant bioinformatics result on NSCLC samples and two cell lines in comparison with NSCLC from the GEO samples is consistent with the aforementioned study (Fig. 5).

**Figure 11:**
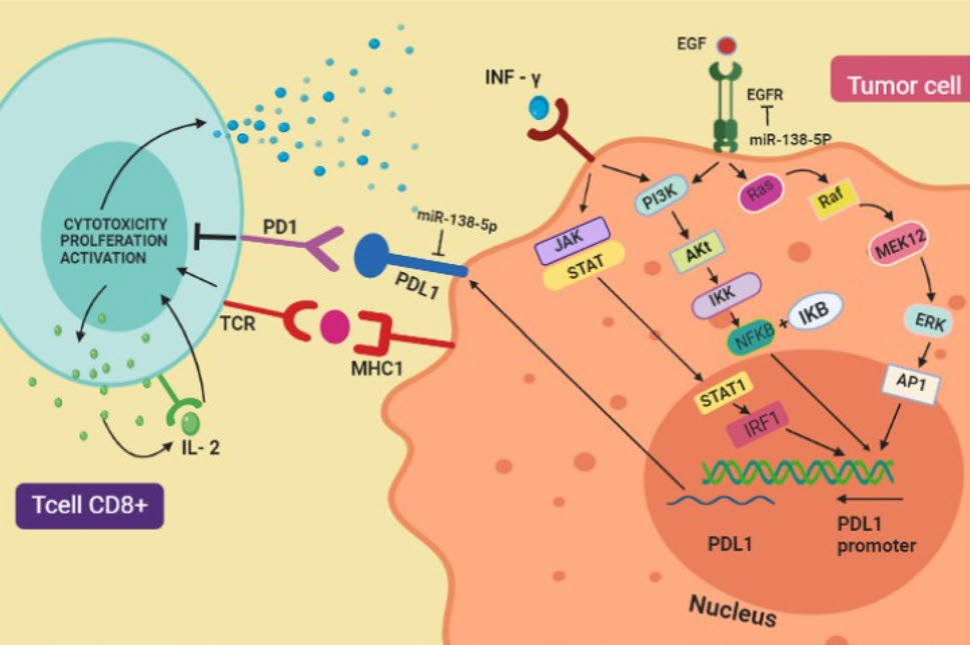
Tumor environmental and tumor-CD8^+^ interaction. The role of self-stimulation and tumor stimulation of activated T cell CD8^+^ through IL-2 and IFN-γ release in the tumor environment revealed that the IFN-γ attaches to its receptor on the surface of tumor cells that contributes JAK-STAT and PI3 kinase signaling pathway. These pathways activate PDL1 expression in the nucleus and then on the surface of tumor cells and PD1-PDL1. The miR-138-5p interrupts the PD1-PDL1 pathway directly (interaction with the PDL1 gene) and indirectly (interaction with the EGFR gene).

Immune checkpoints, such as PD-L1 and PD-1, play a critical role in T lymphocyte dysfunction, attenuation of cytokine secretion, apoptosis, and tumor immune escape by interacting with each other^62^. In the tumor environment, CD8^+^ cells secrete cytokines such as IFN-γ, TNF-α, IL-2, when they encounter the tumor^65^. Cancer cells become aware of the presence of CD8^+^ cells through the IFN-Ƴ receptor on their surface, which induces PDL1 expression on the cancer cell surface, that binds to its ligand (IFN-Ƴ) and activates cancer pathways such as PI3K, Jack-Stat that induces PDL1 expression on the cancer cell surface^66^. They boost PD-L1 in parallel with their twin counterpart (PD-1) level on their surface^67^. T cells induce self-activation by self-stimulation by releasing and receiving IL-2 through their cell surface receptor^68^. Therefore, targeting the PD-1/PD-L1 axis is a promising strategy for cancer immunotherapy (Fig. 11).

The surface expression of PD-1 on Jurak cells and T cells serves as a marker for both exhaustion and over-activation, as demonstrated in previous studies^69,70^. Another study has shown that PHA and IL2 have a stimulatory effect on PD1 expression at the protein level in Jurak cells^71^. For our study, we selected the Jurak human T cell line to represent T cells in tumor-T cell interactions. To simulate the tumor microenvironment, we investigated the effects of specific cytokines (IL-2/PHA and IFN-Ƴ) on the overexpression of PD1 and PDL1 in Jurak cells and A549/Calu6 cell lines, respectively. Our results showed that PDL1 mRNA expression levels were significantly increased by fivefold and sevenfold in both A549 and Calu6 cell lines after 3 days of stimulation with IFN- γ (Fig. 9a). In addition, our study also revealed a significant increase in PD1 expression in Jurak cells after 5 days (64-fold) and 7 days (55-fold) of culturing with media containing PHA and IL-2, as evidenced by both mRNA expression levels (Fig. 9b) and flow cytometry analysis showing over 70% PD1-positive cells (Fig. 9c). To further investigation, the effects of miR-138 on Jurak cell survival, we conducted apoptotic assays under different co-culture conditions. Our results demonstrated that miR-138-5p transduction led to a decrease in Jurak cell mortality when co-cultured with miR-138-5p transduced cell lines (Fig. 10). The inhibition of Jurkat cell mortality when co-cultured with miR-138-5p-transduced Calu6 cells may be indicative of a potential therapeutic effect of miR-138-5p in the treatment of the disease. Further studies may be needed to investigate the underlying mechanisms behind this differential effect and to determine the potential clinical applications of miR-138-5p in the treatment of the disease.

*Potential bias and limitations* The findings of this study have to be seen in light of some limitations. Firstly, our experimental design was focused on two cell lines from the adenocarcinoma subtype of NSCLC containing A549 and Calu6. Both of the cells are from the epithelial tissue of lung cancer and would be great for this comparison. However, if more cell lines of NSCLC from AD (adenocarcinoma) were considered, the results might be broader and more remarkable. Secondly, as a lateral result, we found the close relationships between CD274 and its regulators (some genes in the mRNA-mRNA network) that could be determined in further empirical assays in later studies.

*In conclusion* This study proposes dysregulated miRNAs associated with NSCLC, which will be further investigated in future research. Then, the finding suggests that miR-138-5p may play a role in regulating CD274 expression in NSCLC that inhibits Jurkat cells exhaustion as potential implications for the development of targeted therapies. Additional studies are needed to explore the infrastructural mechanisms of this regulation and its potential clinical significance. Furthermore, our bioinformatics results suggest that CD274 expression may vary between different subtypes of NSCLC, highlighting the importance of considering tumor heterogeneity in the development of personalized treatment strategies. The results also provide valuable insights into the regulation of CD274 expression in NSCLC and identify potential targets (genes with a close relationship with CD274) for future research and therapeutic development.

## Material and methods

**Figure 12:**
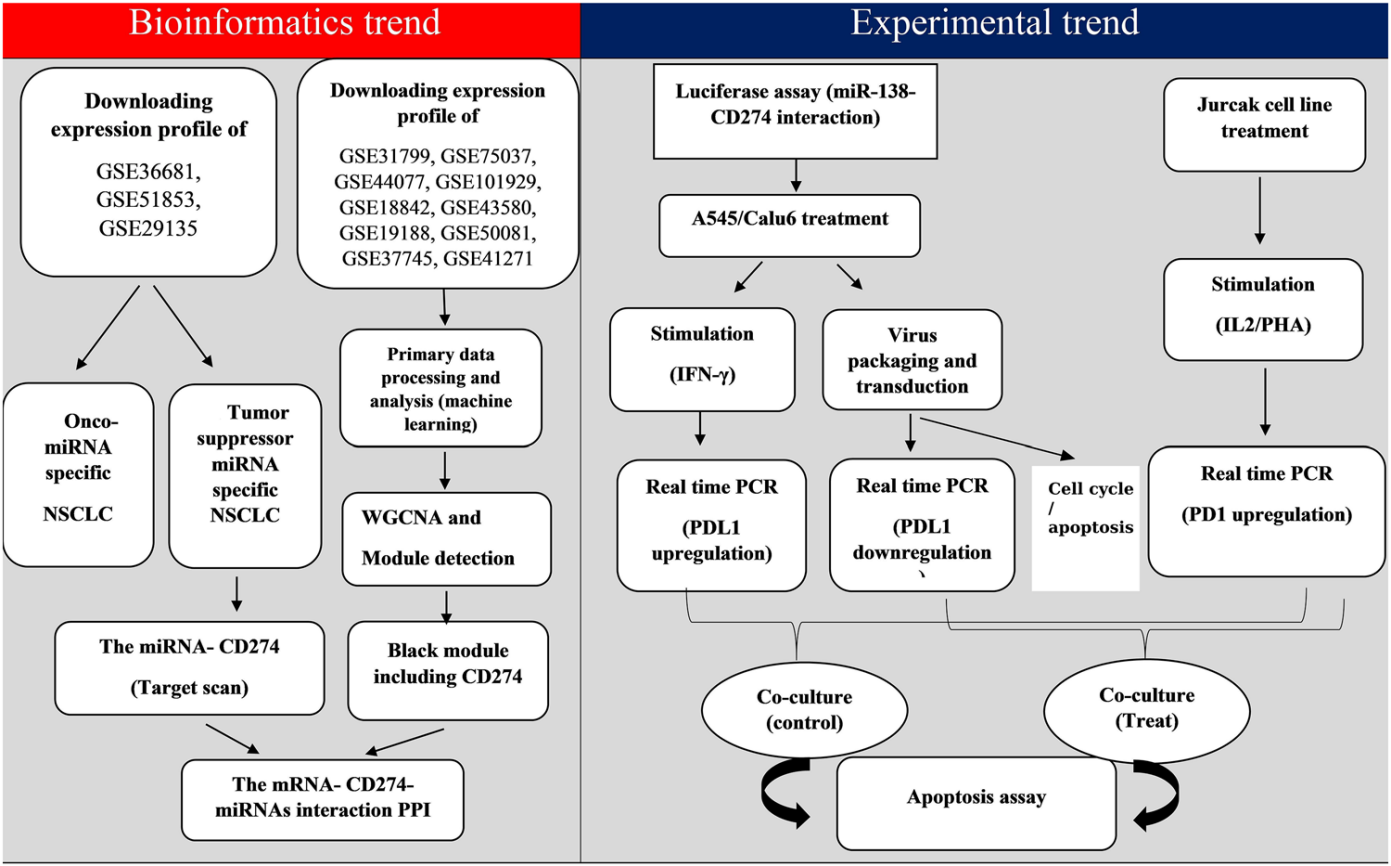
Work flow. The figure represented a glance at the implications.

According to the flow chart (Fig. 12), the material and method are divided into two sections consisting of bioinformatics trends and experimental trends.

### Bioinformatics material and methods

#### Dataset and profiling data

The National Center for Biotechnology Information (NCBI) and the Gene Expression Omnibus (GEO) (https://www.ncbi.nlm.nih.gov/geo) were utilized for downloading Gene expression data from the database population. To gain genes associated with NSCLC, the ten mRNA datasets with GSE31799, GSE75037, GSE44077, GSE101929, GSE18842, GSE43580, GSE19188, GSE50081, GSE37745, and GSE41271 accession were utilized. To obtain miRNA-associated with NSCLC, four miRNA datasets with GSE36681, GSE51853, and GSE29135, GSE169587 accession numbers were used. The information on datasets was located in Supplementary Table S5. Since the aim of our study was to collect information about two common subtypes of NSCLC samples (AC, SCC) to determine specific mRNA and miRNA interactions, the databases including AC and SCC and NSCL samples were selected while some irrelevant samples (Lung carcinoid tumor, airway, Broncho-alveolar and poorly differentiated samples) were removed (Table S6).

#### Preprocessing (normalization and labeling)

All of the miRNA and miRNA datasets were normalized with the GEO query package^72^ in the R programming language. The log2-normal method was implemented for primary normalizing GSE43580 and GSE36681 samples (the value higher than 100) with other mRNA and miRNA datasets, respectively. Moreover, mRNA datasets were merged based on their annotated gene symbols and batch-normalized with the ComBat function available on the SVA package in R Language software to remove batch effect among different datasets. Then, the Normal, Adenocarcinoma and squamous cell carcinoma samples from all datasets were set to Normal, AD, and SCC labels. As the GSE101929 (label: N, T) and GSE18842 (label: tumor, control) datasets have not specific NSCLC subtypes labels, the machine learning classification method includes Support Vector Machine (SVM) from the R Language was used to classify “AD” and “SCC” labels from each other.

#### PCA method (principal component analysis)

The PCA (Principal Component Analysis) method was used for finding data distribution through the first and second principal components and reducing the dimensionality of a data set. PCA aims to identify patterns and probable variations in datasets using the prcomp function in the R language software.

### DEG identification and boxplots

To find fold-change and statistical confidence of selected mRNAs and miRNAs, the Linear Models for Microarray Data (limma)^73^ were used in R language software version 4.1.2. In the limma package, the logarithm of fold change (Log FC) was calculated with the fitting linear model and false discovery rate (FDR), while the *P* value was calculated with moderated t-statistic implemented as common methods to identify differentially expressed genes or miRNAs. The miRNAs and genes were selected using adjusted *P* value using False Discovery Rate (FDR) method (Benjamini and Hochberg's method). The adjusting *P* value is one of the most popular methods for controlling the false discovery rate and making results that are more robust. The boxplots of miRNA and mRNA profiling data were also drawn using geom_boxplot and ggplot function in the ggplot2 R package.

### Network construction and module detection (mRNA)

The co-expression network matrix was constructed by importing all expression data from the combined datasets without performing any preliminary screening of genes or samples using a weighted gene co-expression network (WGCNA)^74^. Based on a hierarchical clustering tree algorithm, we used a TOM dissimilarity measure (1-TOM) to identify block wise gene modules, with at least 100 genes per module. The pick Soft Threshold function is used to choose the soft threshold power of β (β = 7) by considering correlation type (Bicor correlation method) and network type (signed) from the WGCNA package. The Bicor correlation is a statistical method for measuring similarity and association between gene which is less sensitive to outliers making the more significant result. The mergeCloseModules function with mergeCutHeight = 0.6 and minModuleSize = 30 (for merging modules and resulting dendrogram) was used. The module similarity and biological interest modules related to cancerous tissue subtypes were identified by selecting module eigengenes (ME)^75^. Then, the hierarchical clustering method containing cutreeDynamic, plot Dendro and Colors functions in R language software were used for drawing a dendrogram and the correlation between module feature genes.

Finally, the association between modules and traits and P value were determined using ‘Pearson correlation’ (a correlation algorithm), and the t-test student method, respectively. The Pearson correlation Calculates the correlation coefficient between module eigengene and traits (quantitative data) using a linear model that has been used as effect size when studying the relationship between paired data. The *t* test student method with a CorPvalueStudent function in the WGCNA package was used as a hypothesis test to study the difference between two groups of data by Calculating the *P* value (significance) for Pearson correlation results. The effect size for sample groups was calculated using the cohen.d function in effsize package in R language.

### Integration of the PPI Network

The STRING 11.5 database (http://string-db.org)^76^was used for developing the protein–protein interaction (PPI) network (combined score > 0.4) in the selected gene module. The Cytoscape 3.9.1 software (http://www.cytoscape.org/index.html) was applied for protein interaction observation, respectively.

### In silico miRNA/target selection

The miRwalk 2.0 online tool (http://mirwalk.umm.uni-heidelberg.de/) and Target Scan online tool (http://www.targetscan.org/vert-71) were used for predicting microRNAs, which interacted with the CD274 gene.

## Experimental materials and methods

### Dual luciferase reporter assay and vector cloning

Specific primers containing restriction enzyme sites were designed for amplifying mRNA 3′-untranslated regions (3′-UTRs) of CD274 target genes and miR-138-5p by quantitative polymerase chain (PCR were cloned into psiCHECK-2 (add gene, USA) and PCDH-cGFP-Puro-T2A (System Biosciences, CA, USA) vectors, respectively. A dual luciferase assay was performed by seeding the 10^4^ Hek293T cells in a 96well plate (SPL, Germany). After 24 h, 3′-UTRs/control plasmids plus miR-138-5p/scrambled plasmids were co-transfected to seeded Hek293Tcells in a 96-well plate by lipofectamine 3000 reagents (Sigma) in triplicate. Transfected cells were confirmed after 18 h by fluorescence microscope. Then, luciferase activity was measured using the Dual‐Luciferase Reporter assay system kit (Promega, USA) after 56 h. The statistical analysis of the results was performed using a sample T-test test by considering the presentation of standard deviation and P value in the Graph Pad Prism 7.04(San Diego, CA) software.

### Cell culture

The adherent A549, Calu6, and Human embryonic kidney cells (HEK293T) were received from the Stem Cell Technology Research Center (STRC, Tehran, Iran). Cells were cultured in Dulbeccoʼs modified Eagleʼs medium (DMEM) (Gibco, USA), supplemented with 10% fetal bovine serum (Gibco, USA) and Penicillin–Streptomycin, Tissue Culture Grade 1X (Sigma, MO, USA), at 37 °C in an incubator with 95% humidity, and 5% CO2.

The suspended Jurkat cell line was obtained from the STRC and was cultured in Roswell Park Memorial Institute (RPMI) 1640 medium (Gibco, Grand Island, USA), supplemented with 10% fetal bovine serum (Gibco) and Penicillin–Streptomycin, Tissue Culture Grade 1X (Sigma, MO, USA), at 37 °C in the incubator with 95% humidity, and 5% CO2.

The passage number 2 were considered for all adherent cell lines (A549, Calu6, HEK293T) and the Jurkacell line for the rest of the experimental methods.

### Lentiviral packaging and transduction

The PCDH-EF1-CMV-T2A-puro-138-5p and helper vectors (psPAX2and pMD2G-VSVG).

Were co-transfected into seeded HEK293T using Polyethylene imine (PEI, Sigma) reagent. The accuracy of transfection was confirmed using fluorescent microscopy after 16 h of transfection.

Then, the cell supernatants were collected each 12 h for 4 days and stored at 4 °C.

2 × 10^5^ of A549 and Calu6 cells were exposed to 8 ml virus-containing supernatant and 16 μg/ml Polybrene (Sigma), and fluorescent microscopy confirmed the efficiency of transduction at 72 h through GFP detection. The transduced cells were selected using 9 μg/ml puromycin (Sigma) for 72 h, and a flow cytometry assay was utilized to ensure 90% GFP + cells.

### Cell-cycle assay

The adherent A549/Calu6 cells were transduced in a 6well plate (10 × 10^4^ cells/well) with miR-138-5p/scramble viruses. After 72h, both transduced A549/Calu6 cells were harvested and washed with trypsin/ EDTA (Gibco, USA) and PBS (PH7.4, 10 mM). To perform the cell cycle assay, the fixing process was followed by adding the cold 70% ethanol, vortexing, and incubating cells at 4 °C for 4 h before the cell cycle assay. The Ethanol was eliminated by centrifuge (900*g*), and the cells were washed with PBS. After adding RNase (Thermo Fisher Scientific) and incubating for 30 min, the cells were stained by adding a mixture of 200 µl Phycoerythrin (PE; 50 μg/mL)(Sigma), Tryton X-100 (Sigma) and annexin(sigma) incubated for 40 min at 37 °C in the dark environment. Then the cell cycle assay was done, and the result was analyzed using Flow cytometry (BD Biosciences, San Jose, California).

### Adherent and suspended cells stimulation

To stimulate A549/Calu6 cell lines, 5 × 10^5^ of each cell were seeded in a T25 tissue culture flask in Dulbeccoʼs modified Eagleʼs medium (DMEM) (Gibco, Grand Island, USA), supplemented with 10% fetal bovine serum (Gibco) and Penicillin–Streptomycin, Tissue Culture Grade 1X (Sigma, MO, USA), at 37 °C in an incubator with 95% humidity, and 5% CO2. After 24 h, the culture media was washed 3 times with PBS (PH7.4, 10 mM) and exchanged with serum-free media with 15 ng/ml IFNγ for 72 h. To stimulate the Jurket cell line, 2 × 10^5^ Jurkat cells were cultured with RPMI media, supplemented with 10% FBS in a 6well plate. The two wells were considered as a control group, and other wells were stimulated with 5 µg/ml Phytohaemagglutinin M (PHA-M, Gibco, USA) and Human 100 u/ml IL-2 recombinant protein (Gibco, USA) for 5 and 7 days, separately.

### Co-culture of A549/Calu6 with Jurkat cells

To co-cultured A549/Calu6 with Jurkat cells, first, the miR-138-5p/scramble transduced-A549 or—Calu6 cell lines were adopted with RPMI for 10 days. Second, the 5 × 10^5^ each stimulated cells were harvested and seeded into T25 cm2 tissue culture flasks. At confluency of 80%, the medium was changed into fresh medium containing 3 × 10^5^ stimulated Jurkat cells. After 24 h, the flasks were shaken to drive away weekly append Jurkat with culture media into a 15 ml centrifuge falcon. To collect the strongly attached Jurkat cells, the Ethylene diamine tetra acetic acid (EDTA)-free trypsin (Ameresco, Framingham, MA, USA) was added to the flask and incubated until the majority of Jurkat cells were separated without raising the A549/Calu6 cells. After pooling all Jurkat cells from the two mentioned parts, they were centrifuged at 250*g* for 5 min.

### Apoptosis assay

To evaluate the apoptosis rate, the Jurkat cell line co-cultured with transduced NSCLC cell lines was harvested using 500*g* for 5 min. Then, the cells were exposed to 7-AAD-FITC and AnnexinV/PE staining (Bio-Vision, CA) according to the manuscript. Finally, the percentage of early and apoptotic and necrotic cells was analyzed using a BD FACS Calibur flow cytometer (Biosciences, USA).

### Flow cytometry

The stimulated Jurkat samples from all groups were washed with PBS and immune stained with purified APC-conjugated antibody against CD279 (1:200, Biolegend, England) for 60 min at 4 °C in the dark environment, respectively. Then the harvested cells were inquired with FACScan flow cytometry PartecPAS III (Partec, Germany).

### Real-time PCR

Total RNA including miRNAs were extracted from A549 and Calu6 cells 72 h after transduction with miR-138-5p/scrambled using RX Bon (STRC, Iran). Complementary DNA (cDNA) synthesis was conducted using random hexamer and stem-loop primers from the Bon CDNA synthesis kit (STRC, Iran) from the total mRNA and miRNAs, respectively, based on the manufacturer’s instruction. Real-Time PCR was performed with SYBR Green master mix 2X (Ampliqon, Denmark) based on the manufacturer’s instruction via One step (ABI, USA) machine to incubation of reactions at 95 °C for 3 min, followed by 40 cycles of 95 °C for 30 s, and 62 °C for 30 s. The reactions were normalized with HPRT1 and SNORD47 as internal controls for mRNAs and miRNAs, respectively. The primer sequences were located in Supplementary Table S7. All of the experiments were run in triplicate, and the Expression fold changes of miR-138-5p and PDL1 gene were calculated by the 2^–ΔΔCt^ method, respectively.

Then, the fold changes were analyzed statistically using a sample T-test test in the Graph Pad Prism 7.04(San Diego, CA) by considering standard deviation and *P* value.

### Statistical analysis

The Cytoscape software (version 3.9.1) and the R language software were utilized for statistical analysis of the bioinformatics experiments. The bioinformatics statistical methods and their relative steps, association data and functions were categorized in Table 1.

**Table 1:** Statistical analysis.

Step	Associated data	Method	Function
Data preprocessing	Just required on GSE43580 (mRNA) and GSE36681 (miRNA) dataset	Log-2 normalization	To equalize the scale of datasets before merging datasets
	All datasets	Batch normalization	To remove batch differences to merge datasets
		cohen.d function in effsize package in R language	Effect size calculation
		pwr.t.test function in pwr package in R language	Sample size calculation
Finding differentially expressed miRNAs /hsa-miR-138 and CD274 expression levels in GEO samples	Final dataset for mRNA and miRNA datasets	Fitting linear model	To calculate Log FC
		False Discovery Rate (FDR) (Benjamin and Hochberg's method)	To calculate adjusted *P* value
		Moderated t-statistic	To calculate *P* value
Network construction (WGCNA)	Final mRNA dataset	Biweight Midcorrelation (Bicor correlation)	To measure the similarity between genes to find the association among them for constructing WGCNA
Module-trait association (WGCNA)	Module detection	Pearson correlation	To measures the correlation coefficient between quantitative data using a linear model
		t-student Cor *P* value student	To see whether groups are different from each other and provide *P* values to observe the significance

The statistical analysis of the biological results was performed using Graph Pad Prism 7.04 (San Diego, CA) software and Independent (unpaired) samples T-test that is used to compare the means of two groups. The differences between the miR-138-5p-treated group and the scrambled-treated group were determined with a sample T-test by considering the presentation of standard deviation and *P* value (*P* value < 0.05 was considered a statistically significant change).

### Supplementary Information

Supplementary Information.

## Data Availability

The datasets used and/or analyzed during the current study available from the corresponding author on reasonable request.
