# Supplementary material for: PDL1 targeting by miR-138-5p amplifies anti-tumor immunity and Jurkat cells survival in non-small cell lung cancer
Source: Sci Rep. 2024 Jun 12;14:13542. doi: 10.1038/s41598-024-62064-5 (PMC11169246; doi:10.1038/s41598-024-62064-5)
Supplement: Supplementary file 1 — Supplementary Information. [file 41598_2024_62064_MOESM1_ESM.docx]

**PD-L1 targeting by miR-138-5p amplifies anti-tumor immunity and Jurkat cells survival in Non–Small Cell Lung Cancer**

Fatemeh Rostami ^1^, Zahra Tavakol Hamedani^2^, Azadeh Sadoughi^3^, Marzieh Mehrabadi^1^, Fatemeh kouhkan*^1^

1. Department of molecular Biology, Science and Genetic Engineering, Stem Cell Technology Research Center (STRC), Iran university of medical science (IUMS), Tehran, Iran.
2. (Graduated Student) Department of Biotechnology, University of Tehran, Tehran, Iran.
3. Department of Biology, Science and Research Branch, Islamic Azad University, Tehran, Iran.

**Correspondence to:**

*Fatemeh Kohkan, PhD, Associate professor, Stem Cell Technology Research Center,

PO Box: 15856-36473, 15856-36473, Tehran, Iran.


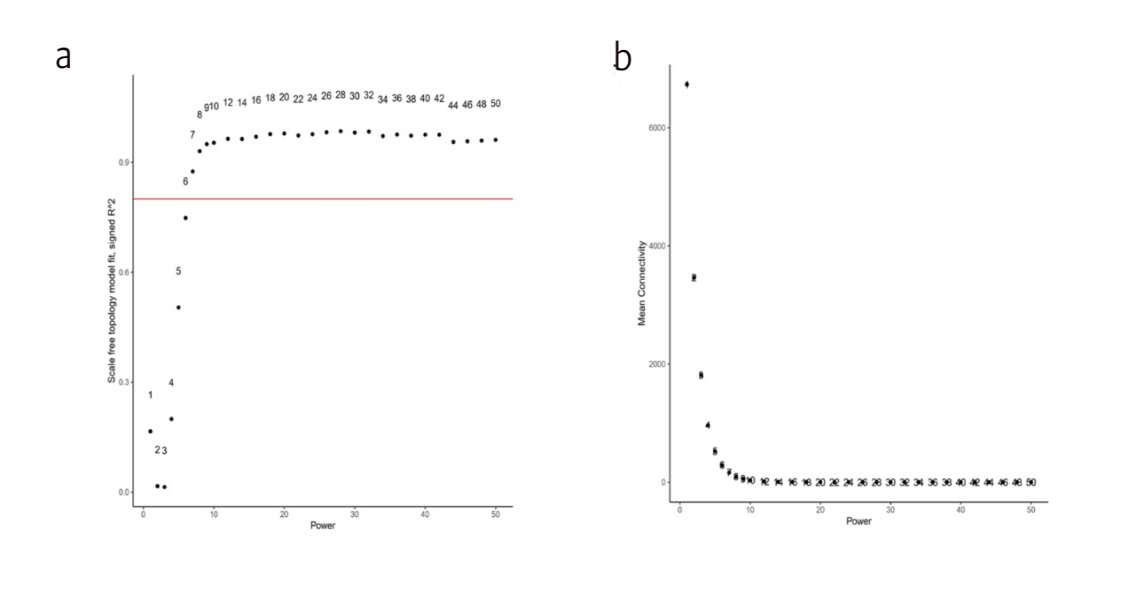


**Figure S1. (a)** Fitting value of adjacency matrix in WGCNA network construction process. The vertical axis is revealed Scale-free topology, while the power of the soft threshold is illustrated on the horizontal axis where it is changing to find the best soft threshold (threshold 7**). (b**) A mean connectivity plot showed the lowest number (7) where the plot is bent.


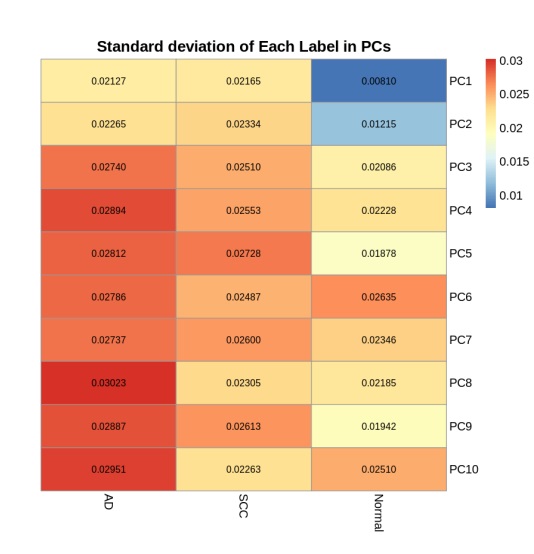


Figure S2. The standard deviation for each label (AD, SCC and Normal samples) across the principal components. In PC1 and PC2, the standard deviation of each sample is smaller compared to other PCs, suggesting that the variation within each label is lesser. Consequently, the labels are more centrally aligned and exhibit greater separation from each other (three separation population).

|  | **logFC (Normal Vs AD)** | **adj.P.Val (Normal Vs AD)** | **Effect Size** | **Lower CI 95%** | **Higher CI 95%** | **logFC (Normal Vs SCC)** | **adj.P.Val (Normal Vs SCC)** | **Effect Size** | **Lower CI 95%** | **Higher CI 95%** |
| --- | --- | --- | --- | --- | --- | --- | --- | --- | --- | --- |
| hsa_miR_218 | -0.82 | 1.90E-05 | 0.5 | 0.3 | 0.71 | -0.68 | 3.90E-05 | 0.63 | 0.38 | 0.88 |
| hsa_miR_522 | -0.7 | 2.00E-05 | 0.52 | 0.31 | 0.72 | -0.62 | 1.50E-05 | 0.6 | 0.35 | 0.85 |
| hsa_miR_34b | -0.59 | 0.009 | 0.36 | 0.15 | 0.57 | -0.55 | 0.0056 | 0.35 | 0.1 | 0.6 |
| hsa_miR_521 | -0.42 | 0.0021 | 0.53 | 0.32 | 0.74 | -0.52 | 1.40E-05 | 0.41 | 0.17 | 0.66 |
| hsa_miR_517c | -0.55 | 0.00072 | 0.44 | 0.24 | 0.65 | -0.51 | 0.00033 | 0.45 | 0.2 | 0.7 |
| hsa_miR_138 | -0.64 | 0.00029 | 0.37 | 0.16 | 0.58 | -0.47 | 0.0032 | 0.53 | 0.29 | 0.78 |
| hsa_miR_133b | -0.78 | 5.80E-06 | 0.38 | 0.17 | 0.58 | -0.45 | 0.0033 | 0.61 | 0.36 | 0.86 |
| hsa_miR_133a | -0.7 | 3.20E-06 | 0.42 | 0.21 | 0.63 | -0.45 | 0.00064 | 0.65 | 0.4 | 0.91 |
| hsa_miR_592 | -0.48 | 0.0039 | 0.32 | 0.11 | 0.52 | -0.37 | 0.013 | 0.38 | 0.14 | 0.63 |
| hsa_miR_206 | -0.3 | 0.033 | 0.36 | 0.16 | 0.57 | -0.35 | 0.0038 | 0.31 | 0.06 | 0.55 |

Table S1: high significant downregulated miRNAs with their features in NSCLC

Table S2: high significant upregulated miRNAs with their features in NSCLC

|  | **logFC (Normal Vs AD)** | **adj.P.Val (Normal Vs AD)** | **Effect Size** | **Lower CI 95%** | **Higher CI 95%** | **logFC (Normal Vs SCC)** | **adj.P.Val (Normal Vs SCC)** | **Effect Size** | **Lower CI 95%** | **Higher CI 95%** |
| --- | --- | --- | --- | --- | --- | --- | --- | --- | --- | --- |
| hsa_miR_183 | 0.66 | 0.0013 | 0.69 | 0.48 | 0.9 | 0.99 | 1.40E-08 | 0.46 | 0.21 | 0.7 |
| hsa_miR_210 | 0.63 | 1.40E-05 | 0.61 | 0.4 | 0.82 | 0.6 | 9.40E-07 | 0.64 | 0.39 | 0.89 |
| hsa_miR_130b | 0.36 | 0.03 | 0.43 | 0.23 | 0.64 | 0.49 | 0.00056 | 0.33 | 0.09 | 0.58 |
| hsa_miR_577 | 0.35 | 0.002 | 0.43 | 0.22 | 0.64 | 0.31 | 0.0014 | 0.45 | 0.21 | 0.7 |
| hsa_miR_9 | 1.2 | 2.30E-05 | 0.36 | 0.16 | 0.57 | 0.7 | 0.0056 | 0.6 | 0.35 | 0.85 |
| hsa_miR_196a | 1 | 0.00022 | 0.34 | 0.14 | 0.55 | 0.68 | 0.0064 | 0.59 | 0.34 | 0.84 |
| hsa_miR_409 | 0.42 | 0.008 | 0.29 | 0.08 | 0.5 | 0.33 | 0.02 | 0.44 | 0.19 | 0.69 |
| hsa_miR_31 | 0.76 | 0.01 | 0.27 | 0.07 | 0.48 | 0.55 | 0.04 | 0.39 | 0.14 | 0.64 |

Table S3: The correlation between modules and phenotypes.

| **Trait** | **Upregulated module** | **Downregulated module** |
| --- | --- | --- |
| Squamous versus Normal samples | Blue (high correlation: 0.79) | Turquoise (high correlation: -0.78) |
| Adenoma versus Normal samples | Brown (Moderate correlation: +0.53) | Black (Moderate correlation: -0.62) |
| Squamous and Adenoma samples | Black (high correlation: 0.72) | Turquoise (Low correlation: -0.34) |

Table S4: The Confidence interval and Effect Size for each trait in mRNA samples

|  | **CD274** | **MYC** | **IL6** | **IL1B** | **IL1A** | **CD58** | **Cd44** | **SERPINB8** |
| --- | --- | --- | --- | --- | --- | --- | --- | --- |
| **logFC (Normal Vs AD)** | 0.602796 | 0.5059 | 1.789174 | 1.260696 | 0.949357 | 0.638613 | 0.537223 | 0.311570 |
| **adj.P.Val (Normal Vs AD)** | 7.10E-19 | 2.60E-11 | 1.75E-42 | 3.78E-41 | 6.31E-27 | 3.33E-42 | 8.70E-29 | 5.27E-13 |
| **Effect Size** | 0.67 | 0.47 | 1.03 | 1.03 | 0.86 | 1.03 | 0.8 | 0.53 |
| **Lower CI 95%** | 0.53 | 0.34 | 0.89 | 0.88 | 0.72 | 0.89 | 0.66 | 0.39 |
| **Higher CI 95%** | 0.81 | 0.61 | 1.17 | 1.17 | 1 | 1.18 | 0.94 | 0.67 |
| **logFC (Normal Vs SCC)** | 0.314420 | -0.4157 | 1.362950 | 0.695838 | -0.039126 | 0.423491 | 0.033977 | 0.041701 |
| **adj.P.Val (Normal Vs SCC)** | 3.39E-05 | 9.04E-07 | 2.09E-21 | 1.16E-11 | 0.709889 | 1.46E-16 | 0.547635 | 0.408650 |
| **Effect Size** | 0.33 | -0.39 | 0.75 | 0.53 | -0.03 | 0.71 | 0.06 | 0.07 |
| **Lower CI 95%** | 0.17 | -0.55 | 0.59 | 0.38 | -0.18 | 0.55 | -0.09 | -0.08 |
| **Higher CI 95%** | 0.48 | -0.24 | 0.9 | 0.69 | 0.12 | 0.87 | 0.21 | 0.22 |
| **logFC (SCC Vs AD)** | 0.288375 | 0.9216 | 0.426224 | 0.564858 | 0.988484 | 0.215122 | 0.503246 | 0.269868 |
| **adj.P.Val (SCC Vs AD)** | 3.84E-06 | 4.18E-39 | 0.000345 | 2.19E-11 | 4.04E-34 | 3.62E-07 | 4.20E-30 | 8.75E-12 |
| **Effect Size** | 0.29 | 0.88 | 0.23 | 0.43 | 0.79 | 0.31 | 0.7 | 0.44 |
| **Lower CI 95%** | 0.17 | 0.75 | 0.11 | 0.3 | 0.66 | 0.19 | 0.58 | 0.32 |
| **Higher CI 95%** | 0.42 | 1.01 | 0.36 | 0.55 | 0.92 | 0.44 | 0.83 | 0.57 |

Table S5: The mRNAs and miRNAs accession numbers with their relative platforms and probes.

| **Datasets Accession Number (in GEO Database)** | **Experiment Type** | **Platform**  **Accession Number (in GEO Database)** | **Number of Probes** |
| --- | --- | --- | --- |
| GSE31799 | Expression profiling by array | GPL14189 | 40102 |
| GSE75037 | Expression profiling by array | GPL6884 | 48803 |
| GSE44077 | Expression profiling by array | GPL6244 | 54675 |
| GSE101929 | Expression profiling by array | GPL570 | 33297 |
| GSE18842 | Expression profiling by array | GPL570 | 33297 |
| GSE43580 | Expression profiling by array | GPL570 | 33297 |
| GSE19188 | Expression profiling by array | GPL570 | 33297 |
| GSE50081 | Expression profiling by array | GPL570 | 33297 |
| GSE37745 | Expression profiling by array | GPL570 | 33297 |
| GSE41271 | Expression profiling by array | GPL6884 | 48803 |
| GSE36681 | Non-coding RNA profiling by array | GPL8179 | 737 |
| GSE51853 | Non-coding RNA profiling by array | GPL7341 | 1146 |
| GSE29135 | Non-coding RNA profiling by array | GPL8179 | 737 |
| GSE169587 | Non-coding RNA profiling by array | GPL25134 | 2549 |

Table S6: The datasets with their Sample Labels

| **Datasets Accession Number (in GEO Database)** | **Sample size** | **Sample labels in each dataset (based on metadata)** | **Final label** |
| --- | --- | --- | --- |
| GSE31799 | 49 | Squamous Cell Carcinoma | SCC |
|  |  | Adenocarcinoma | AD |
| GSE75037 | 166 | Non-malignant | Normal |
|  |  | Adenocarcinoma | AD |
| GSE44077 | 226 | Normal lung | Normal |
|  |  | SCC | SCC |
|  |  | Adenocarcinoma | AD |
|  |  | NSCLC | *Classification using SVM* |
|  |  | Lung carcinoid tumor | *Removed* |
|  |  | airway | *Removed* |
| GSE101929 | 66 | N | Normal |
|  |  | T | *Classification using SVM* |
| GSE18842 | 91 | tumor | *Classification using SVM* |
|  |  | control | Normal |
| GSE43580 | 150 | Squamous Cell Carcinoma stage Ⅰ | SCC |
|  |  | Squamous Cell Carcinoma stage Ⅱ | SCC |
|  |  | Adenocarcinoma stage Ⅰ | AD |
|  |  | Adenocarcinoma stage Ⅱ | AD |
| GSE19188 | 156 | healthy | Normal |
|  |  | AD | AD |
|  |  | SCC | SCC |
| GSE50081 | 181 | Adenocarcinoma | AD |
|  |  | Squamous cell carcinoma | SCC |
|  |  | squamous cell carcinoma | SCC |
| GSE37745 | 195 | Squamous | SCC |
|  |  | Adeno | AD |
| GSE41271 | 275 | Adenocarcinoma | AD |
|  |  | Squamous | SCC |
|  |  | Other labels | *Removed* |
| GSE36681 | 206 | lung adenocarcinoma | AD |
|  |  | uninvolved lung | Normal |
| GSE51853 | 131 | Adenocarcinoma | AD |
|  |  | adenosquamous carcinoma | *Removed* |
|  |  | Large cell carcinoma | *Removed* |
|  |  | Large cell neuroendocrine carcinoma | *Removed* |
|  |  | Squamous cell carcinoma | SCC |
| GSE29135 | 387 | AD | AD |
|  |  | Broncho-alveolar | *Removed* |
|  |  | Adenosquamous | *Removed* |
|  |  | SCC | SCC |
|  |  | Large | *Removed* |
| GSE169587 | 50 | Lung Cancer Tissue | Removed |
|  |  | Lung Normal Tissue | Normal |

Table S7: Primer sequence table.

| **Gene Bank Accession NO** | **Primer name** | **Sequence** | **Annealing Temperature**  **(°C)** |
| --- | --- | --- | --- |
| NM-014143 | CD274(PDL1)-F | 5'-ATC AAG TCC TGA GTG GTA AGA C-3' | 62 |
|  | CD274(PDL1)-R | 5'-GAG GTA GTT CTG GGA TGA CC-3' | 62 |
| NM-005018 | CD279(PD1)-F | 5'-CGTGGCCTATCCATCCTC-3' | 62 |
|  | CD279(PD1)-R | 5'-ATCCCTTGTCCCAGCCACT-3' | 62 |
| NM_000194 | HPRT1 – F | 5'-CCT GGC GTC GTG ATT AGT G-3' | 62 |
|  | HPRT1 – R | 5'-TCA GTC CTG TCC ATA ATT AGT CC-3' | 62 |
| NR_029700 | hsa -miR-138-5p- F | 5'-GCTGGTGTTGTGAATCAGG-3' * | 62 |
| NR_002746 | SNORD47-F | 5'-ATCACTGTAAAACCGTTC-3' * | 62 |

*****Reverse primer for miR-138-5p and SNORD47 purchased from Bonbiotech Company, Iran.
